# Host signaling and EGR1 transcriptional control of human cytomegalovirus replication and latency

**DOI:** 10.1371/journal.ppat.1008037

**Published:** 2019-11-14

**Authors:** Jason Buehler, Ethan Carpenter, Sebastian Zeltzer, Suzu Igarashi, Michael Rak, Iliyana Mikell, Jay A. Nelson, Felicia Goodrum

**Affiliations:** 1 BIO5 Institute, University of Arizona, Tucson, Arizona, United States of America; 2 Vaccine and Gene Therapy Institute, Oregon Health & Science University, Beaverton, Oregon, United States of America; 3 Department of Immunobiology, University of Arizona, Tucson, Arizona, United States of America; State University of New York Upstate Medical University, UNITED STATES

## Abstract

Sustained phosphotinositide3-kinase (PI3K) signaling is critical to the maintenance of alpha and beta herpesvirus latency. We have previously shown that the beta-herpesvirus, human cytomegalovirus (CMV), regulates epidermal growth factor receptor (EGFR), upstream of PI3K, to control states of latency and reactivation. How signaling downstream of EGFR is regulated and how this impacts CMV infection and latency is not fully understood. We demonstrate that CMV downregulates EGFR early in the productive infection, which blunts the activation of EGFR and its downstream pathways in response to stimuli. However, CMV infection sustains basal levels of EGFR and downstream pathway activity in the context of latency in CD34+ hematopoietic progenitor cells (HPCs). Inhibition of MEK/ERK, STAT or PI3K/AKT pathways downstream of EGFR increases viral reactivation from latently infected CD34^+^ HPCs, defining a role for these pathways in latency. We hypothesized that CMV modulation of EGFR signaling might impact viral transcription important to latency. Indeed, EGF-stimulation increased expression of the *UL138* latency gene, but not immediate early or early viral genes, suggesting that EGFR signaling promotes latent gene expression. The early growth response-1 (EGR1) transcription factor is induced downstream of EGFR signaling through the MEK/ERK pathway and is important for the maintenance of hematopoietic stemness. We demonstrate that EGR1 binds the viral genome upstream of *UL138* and is sufficient to promote *UL138* expression. Further, disruption of EGR1 binding upstream of *UL138* prevents the establishment of latency in CD34^+^ HPCs. Our results indicate a model whereby UL138 modulation of EGFR signaling feeds back to promote UL138 gene expression and suppression of replication for latency. By this mechanism, the virus has hardwired itself into host cell biology to sense and respond to changes in homeostatic host cell signaling.

## Author summary

Host signaling is important for regulating states of cytomegalovirus (CMV) replication and latency. We have shown that human cytomegalovirus regulates EGFR levels and trafficking and that sustained EGFR or downstream PI3K signaling is a requirement for viral latency. Changes in host signaling have the ability to alter viral and host gene expression to impact the outcome of infection. Here we show that EGFR signaling through MEK/ERK pathway induces the host EGR1 transcription factor that is highly expressed in hematopoietic stem cells and necessary for the maintenance of hematopoietic stemness. Downregulation of EGR1 promotes stem cell mobilization and differentiation, known stimuli for CMV reactivation. We identified functional EGR1 binding sites upstream of the *UL138* CMV latency gene and EGR1 stimulated *UL138* expression to reinforce the latent infection. Mutant viruses where the regulation of UL138 by EGR1 is disrupted are unable to establish latency in CD34^+^ HPCs. This study advances our understanding of how host signaling impacts decisions to enter into or exit from latency. The regulation of viral gene expression by host signaling allows the virus to sense and respond to changes in host stress or differentiation.

## Introduction

The mechanisms by which herpesviruses persist through the establishment of a quiescent infection, known as latency, and reactivate for continued transmission are incompletely defined. It is known that herpesviruses “sense and respond" to changes in host cell signaling, such as that associated with stress and differentiation, to modulate the “decisions” to maintain latency or to reactivate. However, the molecular underpinnings of how these cellular signals induce changes in chromatin and viral gene expression are less well defined. Human cytomegalovirus (CMV) is a beta-herpesvirus that persists within the majority of the human population. During infection of an immunocompetent host, CMV has a protracted acute phase and then establishes a life-long latent infection, which is marked by sporadic subclinical reactivation events. CMV establishes latency in CD34+ hematopoietic progenitor cells (HPCs) and is carried through differentiation in cells of the myeloid lineage, including CD14+ monocytes [1]. During latency in experimental models, CMV genes are expressed broadly but at very low levels and replication is restricted [2, 3]. Reactivation in immunodeficient individuals, such as stem cell or solid organ transplant recipients, is a major cause of morbidity and mortality [4–6]. Additionally, CMV reactivation in patients undergoing intensive chemotherapy treatments can cause severe disease, including pneumonia, enteritis, blindness, and deafness[7, 8]. Currently, there is no vaccine and existing antivirals are limited by toxicity and cannot target latently infected cells. Understanding the molecular mechanisms that define and control the latent CMV infection is critical for the development of novel strategies to target the latent infection.

Virus manipulation of host cell signaling during infection of hematopoietic cells provides the means by which CMV ensures the survival of the infected cells and controls differentiation and reactivation [9–14]. Epidermal growth factor receptor (EGFR) signaling is a key component of the molecular switch regulating the establishment of latency and reactivation of viral replication [15]. In CD34+ HPCs where the virus establishes latency, CMV stimulates EGFR during entry and these initial signaling events are important for the establishment of latency [16]. Inhibition or EGFR or downstream PI3K signaling increases replication in fibroblasts and reactivation in CD34+ HPCs, suggesting important roles for EGFR signaling in CMV infection. As such, EGFR is an interesting point of control for virus manipulation.

The CMV UL135 and UL138 gene products antagonize one another in regulating latency and reactivation [17]. UL138 is suppressive to virus replication and critical for the establishment of latency, whereas UL135 is important for reactivation. UL135 functions, in part, by overcoming the suppressive effects of UL138, which otherwise block the initiation of viral replication from infectious genomes. Accordingly, UL135 and UL138 gene products both interact with EGFR, but have opposing effects on the regulation of EGFR trafficking and signaling [15]. UL138 sustains EGFR signaling, whereas UL135 reduces total and cell surface levels of EGFR to attenuate signaling. UL135 regulates EGFR trafficking and signaling through its interactions with the host adapter proteins for the Cbl E3 ubiquitin ligase, Abelson interacting protein 1 (Abi1) and CIN85/CD2AP [15, 18]. Mutations in *UL135* ablating these host interactions restore EGFR levels in infected cells and diminish reactivation from latent infection [18]. The requirement for UL135 interaction with Abi-1 and CIN85/CD2AP for reactivation, directly links UL135-mediated degradation of EGFR to reactivation [18].

In this study, we investigated the role of signaling downstream of EGFR in productive infection in fibroblasts and in latency in CD34+ HPCs. CMV infection blunts EGFR signaling and downstream pathways in productive infection, but EGFR signaling and that of downstream pathways is sustained in CD34+ HPCs. Virus replication and reactivation were enhanced in CD34+ HPCs when MEK/ERK, STAT, and particularly PI3K pathways downstream of EGFR were inhibited. EGF-stimulation of infected fibroblasts increased UL138 gene expression, suggesting that EGFR signaling impacts UL138 gene expression. We mapped consensus binding sites for the early growth response factor 1 (EGR1) transcription factor upstream of *UL138*. EGR1 is induced downstream of EGFR, is highly expressed in CD34+ HPCs and is required to maintain stemness [19–21]. Here, we show that EGR1 binds to sites within the *UL133-UL138* gene locus (UL133/8) and stimulates UL138 gene expression for latency in CD34^+^ HPCs. From these findings a positive feedback model emerges whereby UL138 sustains EGFR signaling and EGFR signaling stimulates EGR1, which feeds back to drive UL138 gene expression. Disruption of EGR1 regulation of UL138 expression results in a loss of latency and the virus replicates. The regulation of UL138 gene expression by host signaling provides a mechanism by which the virus senses and responds to changes in host stress or differentiation. Our findings advance our understanding of how host signaling impacts the decisions to enter into or exit from latency.

## Results

### Explanation and integration of model systems

CMV infects a diverse array of cell types in the host. Important insights into CMV infection are gleaned from using and integrating multiple model systems. While CMV productively replicates to varying extents in a large number of cells, including fibroblasts and epithelial cells, latency is thought to be restricted to hematopoietic progenitor cells and cells of the myeloid lineage, including monocytes [14]. CMV can be detected in hematopoietic cells as far back in the hierarchy of hematopoietic differentiation as CD34+ progenitor cells [1, 22, 23] and primary CD34+ HPC models have become the gold standard for understanding CMV latency. However, the scope of experiments using CD34+ HPCs is limited by their heterogeneity, scarcity in the blood and bone marrow, availability, and poor amenability to molecular approaches, including expression of transgenes or knockdown. Therefore, we use both fibroblasts and CD34+ HPCs in our studies to understand how the virus manipulates host pathways and the significance of this biology to infection. Indeed, the function of viral genes that modulate the decision to maintain or exit latency are in large part conserved across cell systems, it is the impact of their function that differs depending on the context. For example, UL138 is suppressive to virus replication across cell systems. In CD34+ cells, disruption of UL138 results in a replicative virus that fails to establish latency [24, 25]. In productive infection of fibroblasts, the suppressive effects of UL138 are overridden by high levels of gene expression and the action of UL135 but become readily apparent when UL135 is disrupted [17]. Our use both fibroblasts and CD34+ HPCs in these studies permits us to apply a broad array of methods to develop an in depth understanding of the biology of infection in multiple contexts.

### CMV downregulates total and cell surface levels of EGFR

**Fig 1:**
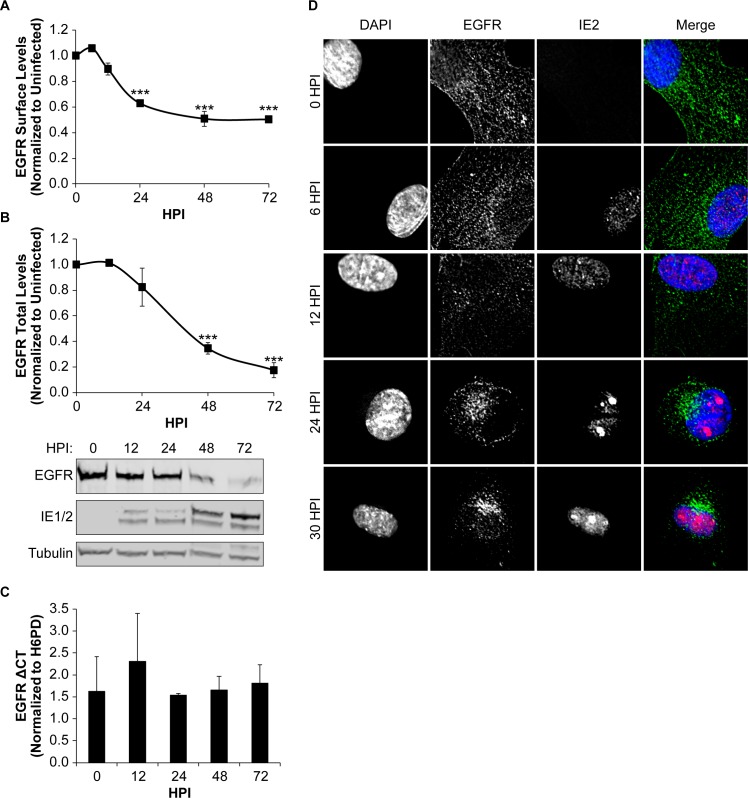
CMV downregulates EGFR surface and total protein levels as infection progresses. Fibroblasts were infected with TB40/E_GFP_ virus at an MOI of 1 for 0–72 hpi, with 0 hpi being uninfected. (A) To measure EGFR surface levels, infected cells were stained with brilliant violet 421 conjugated α-EGFR antibody and analyzed by flow cytometry. Normalized geometric mean fluorescent intensity is shown. (B) Total EGFR levels were measured over a time course by immunoblotting. Blots were stained with α-EGFR, α-IE1/2 antibody, and α-Tubulin. Both surface (A) and total (B) EGFR levels were normalized to 0 hpi for statistical analysis. IE proteins serve as a control for infection and tubulin serves as a control for loading. (C) Relative EGFR mRNA levels were measured over a time course using quantitative reverse transcriptase PCR and SYBR green. EGFR transcripts are normalized to H6PD, cellular housekeeping control, at each timepoint. (A-C) Statistical significance was calculated by One-Way ANOVA with Tukey’s correction and represented by asterisks (*** p-values < 0.001). Graphs represent the means from 3 independent replicates with error bars representing SEM. (D) Subcellular localization of EGFR was monitored over a time course of infection. Nuclei, EGFR and IE2 are visualized by staining with DAPI, α-EGFR, and α-IE2 and confocal deconvolution microscopy.

We previously demonstrated that CMV modulates EGFR total and cell surface levels during infection in fibroblasts (productive infection) and CD34^+^ HPCs (site of latency)[15]. In fibroblasts, EGFR surface and total levels decrease substantially by 48 hours post infection (hpi). To further understand the regulation of EGFR during productive infection, we analyzed surface and total levels of EGFR in fibroblasts over a time course of infection from 0–72 hpi. We measured surface levels of EGFR in fibroblasts infected with the TB40/E strain expressing GFP as a marker for infection [26], which serves as the parental/wild-type (WT) virus for all studies (Fig 1A). EGFR surface levels began to decrease by 12 hpi and were reduced to ~60% of uninfected (0 hpi) cells by 24 hpi, and remained between 50 and 60% of uninfected cells for the remainder of the infection time course. Analysis of total EGFR levels over the same time course indicated that total EGFR levels were reduced to 40% and 20% of uninfected cells by 48 and 72 hpi, respectively (Fig 1B). These findings are consistent with our previous work demonstrating the downregulation of EGFR during the productive cycle of infection [15, 18] and extend those observations by defining the onset of this downregulation as within the early stages of infection.

CMV was previously shown to transcriptionally downregulate EGFR [27, 28]. While UL135 downregulates total levels of EGFR, disruption of UL135 or its interaction with host proteins does not fully restore EGFR to uninfected cell levels [15, 18]. This might be explained by transcriptional downregulation of EGFR in addition to the targeted UL135-mediated turnover of EGFR. However, we did not detect any significant alteration in EGFR mRNA levels by quantitative reverse transcriptase PCR (RT-qPCR) over the time course of infection(Fig 1C). The lack of a transcriptional downregulation in our study may reflect differences in the virus strain used for infection or the cell type, and leaves open the possibility that other viral factors contribute to the diminishment of EGFR levels.

We previously observed a re-localization of activated EGFR to the juxtanuclear viral assembly compartment during productive replication [15]. To determine the timing of EGFR re-localization, EGFR subcellular localization was determined in TB40/E infected fibroblasts at 0–30 hpi(Fig 1D). IE2 staining marked infected cells. In uninfected cells (0 hpi), EGFR staining is localized at the plasma membrane and distributed in puncta throughout the cytoplasm. By 24 hpi, a large portion of EGFR was localized to the juxta-nuclear region and maintained there. We previously demonstrated that this juxta-nuclear localization is proximal to markers, GM130 and pp28, for the viral assembly compartment [15]. This result indicates that EGFR is re-localized at early times in infection prior to the formation of the assembly compartment, which does not become evident until >48 hpi.

### EGFR and downstream pathways are inhibited by CMV as replication progresses

To determine how CMV productive infection impacts EGFR signaling and pathways downstream of EGFR, we analyzed phosphorylation of EGFR, MEK1/2, ERK1/2, STAT3, PLCγ, and AKT at steady state or following 30 min of EGF stimulation in infected fibroblasts using a EGFR signaling array (PathScan, Cell Signaling Technologies). At steady state, phosphorylation of EGFR at T669, Y845, and Y1068 was increased by CMV infection relative to uninfected cells. However, EGFR was less responsive to EGF stimulation, marked by decreased phosphorylation on T669, Y845, and Y1068 relative to uninfected, stimulated cells (S1 Fig). Infection did not alter phosphorylation of EGFR Y998. While basal activity of MEK1/2, ERK1/2, and AKT was not altered by infection in unstimulated cells, EGF-stimulated phosphorylation of MEK1/2 (S221 or S217/221) and AKT (S473) was reduced in infected cells relative to uninfected cells (S1 Fig). CMV infection did not affect phosphorylation and activation of STAT3 (Y705), PLCγ1 (S1248) or AKT (T308) in response to EGF stimulation (S1 Fig). These results suggest that MEK/ERK and AKT signaling pathways downstream of EGFR are blunted by CMV infection.

**Fig 2:**
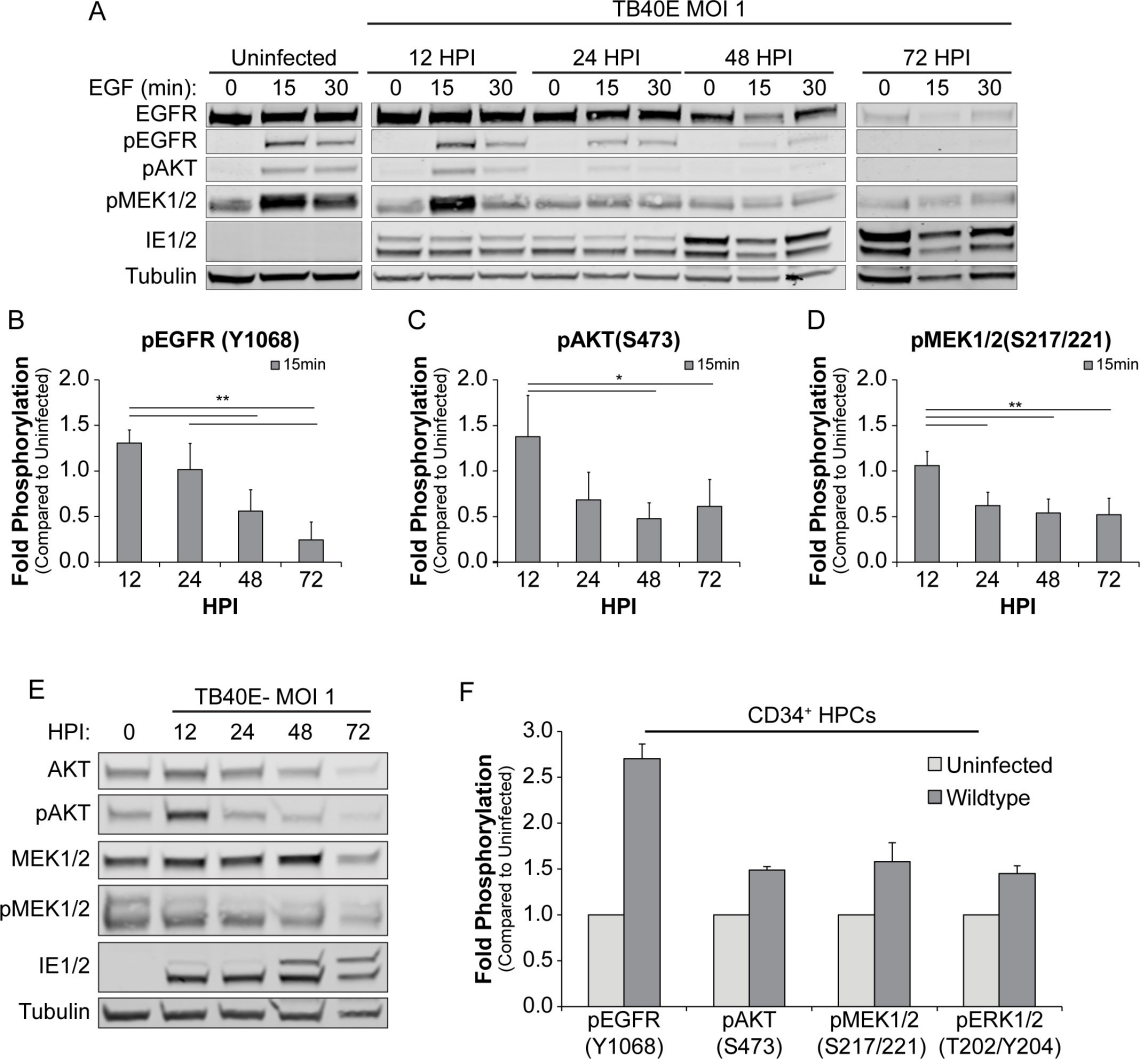
CMV infection prevents activation of AKT and MEK1/2. (A) Fibroblasts were serum starved for 24h and cells were then infected for 0–72 hpi. At each timepoint, infected cells were pulsed with 10 nM of EGF for 0–30 min, and lysed. Lysates were separated out on SDS-PAGE gel, transferred on PVDF membrane, and stained for α-EGFR, α-pEGFR (Y1068), α-pAKT (S472), α-pMEK1/2 (S217/221), α-IE1/2 antibody, and α-Tubulin. (B) The 15 min post EGF timepoint for all phosphorylation markers were normalized to uninfected cells and graphed to calculate statistics. Statistical significance was calculated by One-Way ANOVA with Tukey’s correction and represented by asterisks (* p-value < 0.05 and ** p-value < 0.01).Graphs represent the mean of three replicates and error bars represent SEM. (E) Lysates from the 15 min post EGF-pulse at each time point post infection was separated by SDS-PAGE, transferred on PVDF membrane, and stained for α-AKT, α-pAKT(S472), α-MEK1/2, α-pMEK1/2(S217/221), α-IE1/2 antibody, and α-Tubulin to analyze total levels of AKT and MEK1/2. (F) CD34^+^ cells uninfected or infected with WT CMV (MOI of 2) were fixed and permabilized at 48 hpi. Cells were stained with PE conjugated α-CD34^+^, Alexa Fluor 350 conjugated α-pEGFR(y1068), Dylight 649 conjugated α-pAKT(S473), Alexa Fluor 647 conjugated pMEK1/2(S217/221), and PerCP-eFluor 710 conjugated pERK1/2(T202/Y204) and the geometric mean of fluorescence determined by flow cytometry. Bars represent the average fold change in the geometric means from two replicates. Error bars represent the range of the two replicates.

To further analyze the inhibition of both AKT and MEK1/2 pathways by CMV, we monitored their responsiveness to EGF over a time course of infection. Serum starved, infected fibroblasts were pulsed with EGF ligand at 0, 12, 24, 48, and 72 hpi and cell lysates were harvested at 0, 15 and 30 minutes following each EGF pulse to analyze the phosphorylation of EGFR (Y1068), AKT (S473), and MEK1/2 (S217/221) (Fig 2A). In uninfected fibroblasts, EGFR, AKT and MEK1/2 phosphorylation was induced by 15 min post EGF stimulation, as expected. In fibroblasts infected for 12 hours, pEGFR, pAKT, pMEK1/2 induction was unchanged from uninfected cells. In contrast, the levels of all three phosphorylation markers decreased significantly after EGF stimulation with a reduction of pMEK1/2 by 24 hpi and both pEGFR and pAKT by 48 hpi, relative to uninfected fibroblasts (Fig 2B). By 72 hpi, pEGFR and pAKT was undetectable in infected cells (Fig 2A). Induction of pMEK1/2 in response to EGF stimulation is undetectable relative to basal levels by 24 hpi. Total levels of AKT and MEK1/2 (Fig 2E) did not change over the course of infection until 72hpi. Together these data indicate that while infection sustains or induces basal levels of EGFR signaling at early times, EGFR and both the MEK/ERK and PI3K/AKT downstream signaling nodes become progressively less responsive to stimulation in the productive infection. These results are consistent with the reduction of EGFR from the cell surface during early times in infection.

In the context of CD34+ HPCs and in contrast to fibroblasts, surface levels of EGFR are sustained or induced by CMV infection [15]. However, similar to infection in fibroblasts, basal levels of phosphorylated EGFR and downstream pathways were sustained or increased by CMV infection (PathScan, S1E–S1H Fig). Using phosphoflow, we find that EGFR (Y1068), AKT (S473), MEK1/2 (S217/221), and ERK1/2 (T202/Y204) were induced or sustained following infection with CMV at 2 dpi (Fig 2F). Due to the levels low levels of EGFR on the surface of CD34+ HPCs, limitations of cell numbers, and the inability to serum starve, we are not able to analyze signaling in CD34+ HPCs following an EGF pulse.

### Pathways downstream of EGFR suppress viral replication for latency

**Fig 3:**
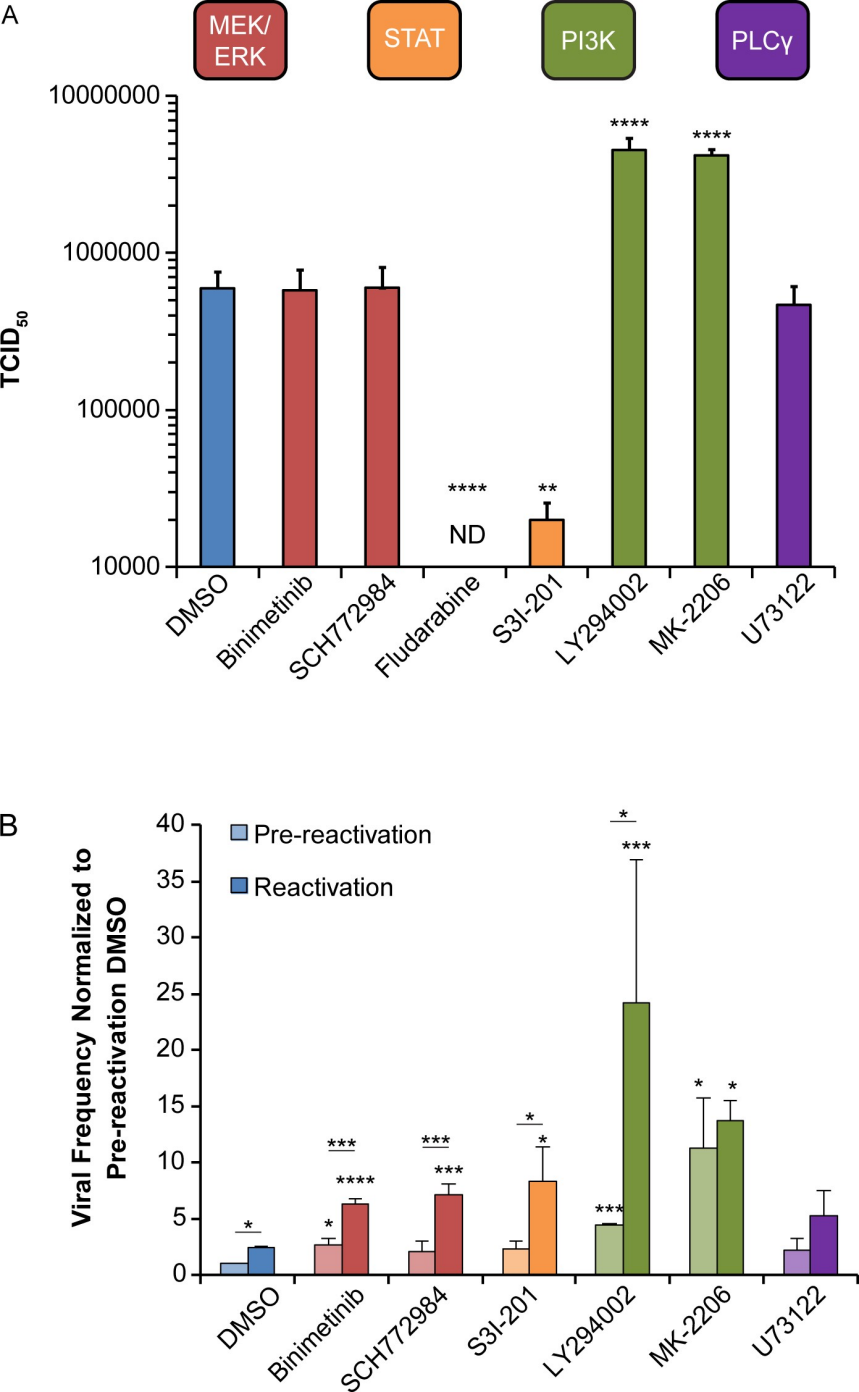
Inhibition of MEK/ERK and PI3K/AKT signaling stimulates reactivation in CD34^+^ HPCs, but only inhibition of PI3K/AKT stimulates replication in fibroblasts. (A) Fibroblasts were infected with TB40/E_GFP_ virus (MOI = 1). At 24 hpi, cells were treated with DMSO (vehicle control), MEK/ERK (Binimetinib 1 μM; SCH772984 125nM), STAT (Fludarabine 50 μM; S3I-201 100 μM), PI3K/AKT (LY294002 20 μM; MK-2206 1.25 μM), or PLCγ (U73122 4 μM) inhibitors. At 8 dpi media and cells were collected and viral titers were determined by TCID_50_. (B) CD34^+^ HPCs were infected with TB40/E_GFP_ virus (MOI = 2). At 24 hpi, CD34^+^/GFP^+^ cells were sorted and put into long-term culture with inhibitors listed above. After 10 days, parallel populations of either mechanically lysed cells or whole cells were plated onto fibroblasts monolayers in cytokine-rich media and frequency of infectious centers was determined by limited dilution analysis. The mechanically lysed population defines the quantity of virus present prior to reactivation (pre-reactivation). The whole cell population undergoes differentiation due to fibroblasts contact and cytokine stimulation, which promotes viral reactivation (reactivation). The frequency was normalized to the pre-reactivation DMSO control to facilitate comparisons between experiments. Statistical significance was calculated by either One-Way ANOVA with Bonferroni(A) or Two-Way ANOVA with Tukey’s correction for each condition (B) and represented by asterisks (* p-value < 0.05, ** p-value < 0.01, *** p-value < 0.001, and **** p-value < 0.0001). For fludarabine infected fibroblasts ANOVA could not measure difference due to absence of quantifiable virus and statistical significance was calculated by student t-test (**** p-value < 0.0001). Data graphed is the mean of 3 replicates with error bars representing SEM.

Work by the Chan and Yurochko groups have demonstrated that PI3K signaling is important to survival of CMV-infected monocytes [10, 29]. Further, we previously demonstrated that inhibition of EGFR or PI3K increases CMV replication in fibroblasts and reactivation in CD34^+^ HPCs [15]. To further investigate how pathways downstream of EGFR impact CMV replication and latency, we used chemical inhibitors of the MEK/ERK, STAT, PI3K/AKT, and PLCγ pathways. The efficacy of each inhibitor at the chosen concentration was confirmed by analyzing phosphorylation over a 5-day time course in fibroblasts (S2 Fig). We also analyzed treated cells for viability at 5 dpi (S3 Fig). Note that MK-2206 is not shown due to autofluorescence associated with the inhibitor in this assay that precluded the accurate quantitation of live cells; however, cell death is of little concern with this drug given the high levels of virus replication in treated cells. Fibroblasts were treated with inhibitors at 1 day post infection so as not to interfere with viral entry [16, 30, 31] and viral titers were measured at 8 dpi (Fig 3A). Inhibition of PI3K (LY294002) or AKT (MK-2206) increased viral titers by 7.6 and 7-fold, respectively, in comparison to the vehicle control, similar to what we have previously reported with EGFR inhibition [15]. In contrast, inhibition of STAT1 (Fludarabine) or STAT3 (S3I-201) decreased virus production. Loss of viral replication by STAT inhibition has previously been reported with these and similar inhibitors [32, 33] and is not due to a loss in cell viability (S3 Fig). Inhibition of MEK1/2 (Binimetinib), ERK1/2 (SCH772984), or PLCγ (U73122) did not alter virus titers relative to the vehicle control. These data confirm that PI3K/AKT pathways are suppressive to virus replication and demonstrate that MEK/ERK and PLCγ pathways are dispensable for productive infection in fibroblasts.

To determine the importance of the signaling pathways downstream of EGFR to latency and reactivation, we analyzed latency and reactivation in CD34+ HPCs infected in vitro and treated with inhibitors to each pathway. Infected (GFP+) CD34^+^ HPCs were isolated by fluorescent activated cell sorting (FACS) and co-cultured with or without indicated inhibitors for 10 days in long-term bone marrow cultures using a bone marrow stromal cell support that has been shown to maintain hematopoietic cell progenitor phenotype and function [34]. This period in long-term bone marrow culture allows for the establishment of CMV latency. At 10 dpi, half of the cells were seeded by limiting dilution into co-culture with fibroblasts in a cytokine-rich media to promote myeloid cell differentiation and reactivation. The other half of the culture was lysed and seeded by limiting dilution in parallel onto fibroblasts to quantify virus formed during the latency period (pre-reactivation) [35]. Reactivation resulted in a 2–3 fold increase in the frequency of infectious centers relative to the pre-reactivation control (DMSO control, Fig 3B). Inhibition of MEK, ERK, or STAT increased the frequency of infectious centers by at least 2-fold in both the pre-reactivation and reactivation condition relative to their respective DMSO control. Inhibition of PI3K (LY294002) induced a 4-fold increase in infectious centers in the pre-reactivation and an almost 10-fold increase in infectious centers in the reactivation. Similarly, inhibition of AKT (MK-2206) resulted in a 10-fold increase in the frequency of infectious centers in the pre-reactivation and a 5.5-fold increase in the reactivation, relative to DMSO controls. By contrast, inhibition PLCγ did not significantly alter infectious centers produced prior to or following reactivation relative to the DMSO controls. We monitored proliferation of infected, drug treated CD34+ HPCs over the 10-day culture period to ensure that the drug treatments did not result in a loss of cell viability as indicated by continued proliferation (S3 Fig). Fludarbine was not used in CD34+ HPCs because a non-toxic dose could not be found. These results indicate that the MEK/ERK, STAT and PI3K/AKT pathways each contribute to the maintenance of CMV latency and inhibition of these pathways enhances reactivation.

### EGF stimulation drives expression of the *UL138* latency determinant

Collectively, our work demonstrates a requirement for EGFR and its downstream MEK/ERK, STAT, and PI3K/AKT pathways for the suppression of virus replication to maintain latency in CD34+ (Fig 2B) [15]. However, the mechanisms by which host signaling impacts infection is not known. While the effect of EGFR signaling on cellular gene expression [36] might impact virus replication and latency, we hypothesized that EGFR signaling might also impact viral gene expression and, specifically, the expression of genes required for latency.

**Fig 4:**
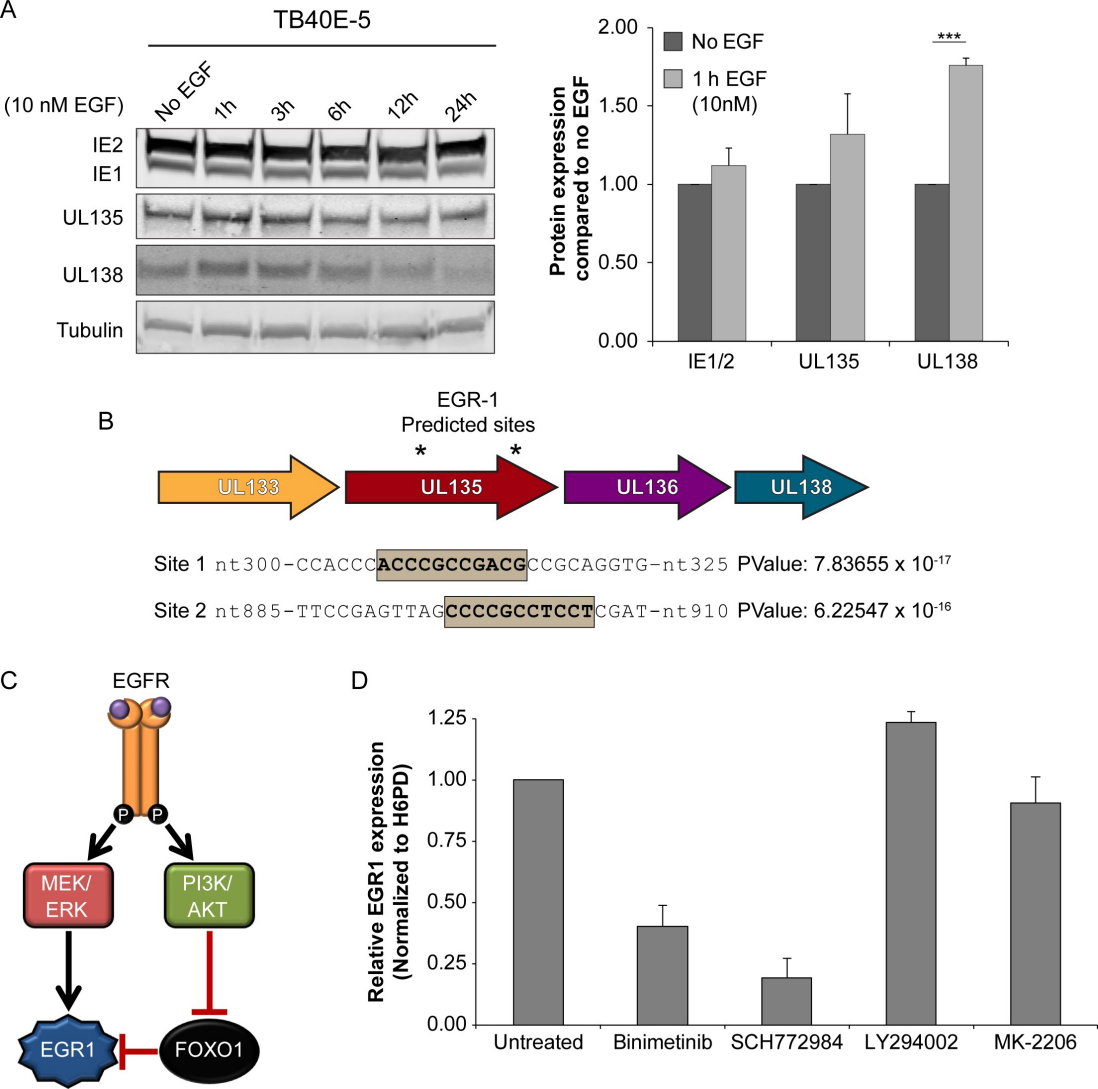
EGFR signaling promotes UL138 protein accumulation. (A) Fibroblasts were infected with TB40/E_GFP_ (MOI = 1) and put into serum-free media at 24hpi. Cells were stimulated with 10nM EGF at 48 hpi and cells were harvested between 1 and 24 hours post stimulation. Lysates were separated by SDS-PAGE, transferred and blotted with α- IE1/2, α-*UL135*, α-*UL138*, and α Tubulin. Protein levels from 4 replicates were normalized to no EGF treated control and 1h post EGF treatment is graphed. Statistical significance was calculated by student t-test; asterisks *** p-value < 0.001. Error bars represent SEM.(B) Graphical representation of putative EGR1 binding sites located within *UL135* ORF starting at nt-306 and nt-896, in reference to UL135 start codon. P-values were calculated using PhysBinder prediction software. (C) A model depicting how EGFR signaling promotes EGR1 expression by either directing its expression through MEK/ERK signaling or by blocking FOXO1 suppression of EGR1 transcription though PI3K/AKT signaling. (D) CD34^+^ HPCs were infected with WT virus (MOI of 2) and treated with MEK/ERK (Binimetinib and SCH772984) or PI3K/AKT (LY294002 and MK-2206) inhibitors at 4 hpi. RNA was isolated at 48 hpi EGR1 measured by RT-qPCR relative to H6PD. Graph represents the average from two replicates with error bars that represent the range.

To determine if stimulation of EGFR might affect expression from the *UL133-UL138* locus, we monitored protein accumulation from the immediate early *UL122* and *UL123* (IE2 and IE1, respectively), *UL135*, and *UL138* gene expression in serum starved, infected fibroblasts over a time course following EGF stimulation (Fig 4A). UL138 protein accumulation increased by 75% at 1h following EGF stimulation relative to unstimulated cells. UL138 protein levels remained elevated for up to 6h post EGF pulse. In contrast, neither *UL135* nor IE1/2 levels changed in response to EGF-stimulation. These results indicate that EGFR signaling inflences expression of the UL138 latency determinant.

To begin to understand how EGFR signaling might affect *UL138* expression, we used PhysBinder to identify putative transcription factor binding sites within the *UL133-UL138* locus that are regulated by EGFR signaling [37]. To minimize the potential for false positives, we used the Max Precision setting and identified two binding sites for early growth response factor 1 (EGR1) within the *UL135* open reading frame (ORF) upstream of *UL138* (Fig 4B). EGR1 is highly expressed in CD34+ HPCs and is required to maintain stem cell quiescence and retention in the bone marrow [19, 38]. The downregulation of EGR1 is required for differentiation of stem cells and migration out of the bone marrow [19]. EGR1 expression can be induced by both the MEK/ERK and PI3K/AKT pathways downstream of EGFR (Fig 4C) [39–41]. To determine if EGR1 is regulated through pathways downstream of EGFR in infected CD34+ HPCs, we treated WT-infected cells at 4 hpi with inhibitors to MEK, ERK, PI3K or AKT for 2 days and then quantified EGR1 transcripts by RT-qPCR. Intriguingly, inhibition of MEK/ERK, but not PI3K/AKT reduced EGR1 transcript levels (Fig 4D), suggesting that EGR1 levels in CMV infected CD34+ HPCs depend upon MEK/ERK signaling.

### EGR1 binds sequences upstream of UL138

**Fig 5:**
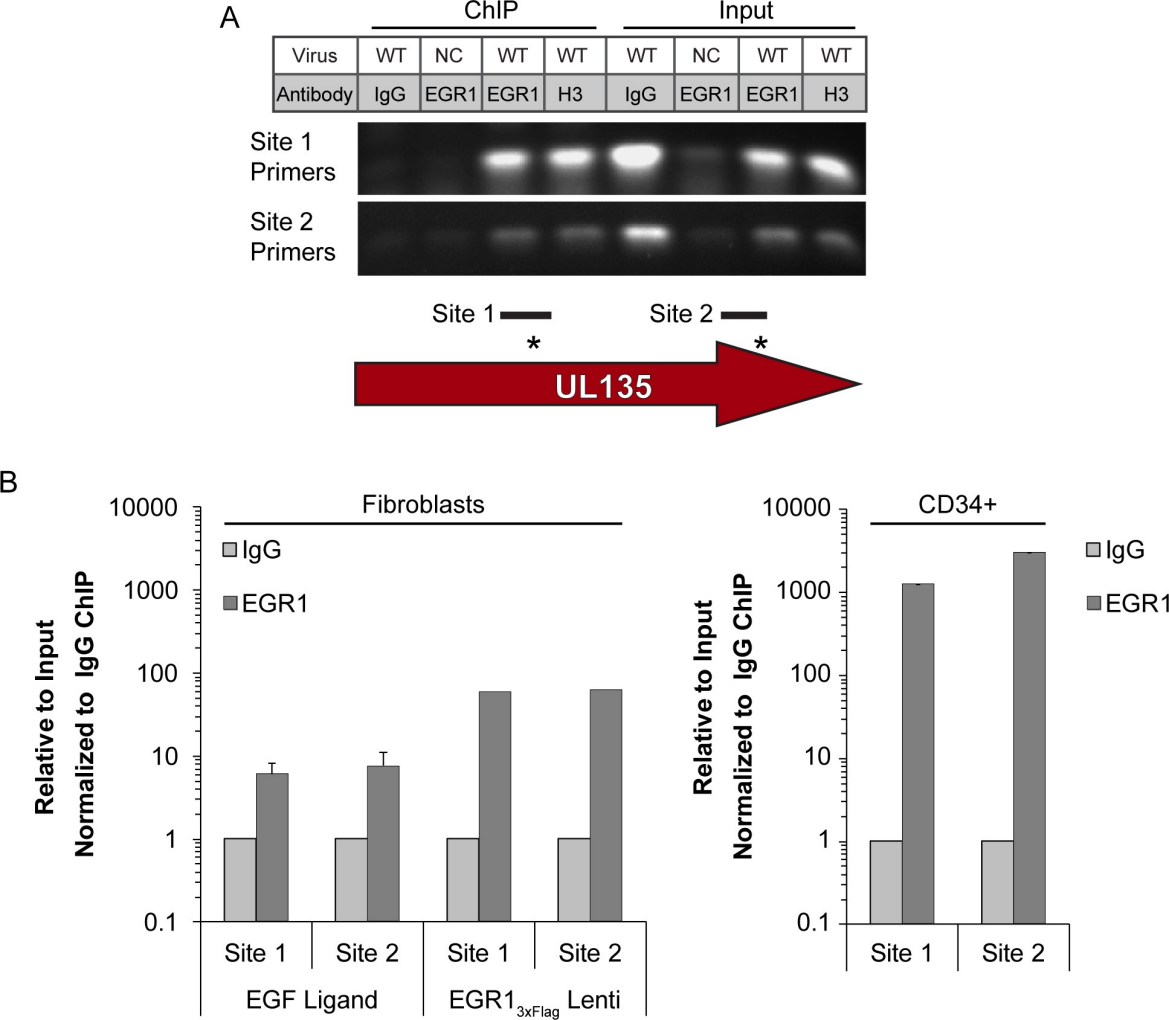
EGR1 transcription factor binds within the *UL135* gene region. (A) Fibroblasts were transduced with EGR1_3xFlag_ lentivirus and then infected with WT or *UL133/8*_*null*_ mutant (negative control; NC) TB40/E virus (MOI = 1). Chromatin was immunoprecipitated (ChIP) with IgG control or antibodies specific to EGR1 or histone 3 (H3) and the presence of Site 1 or Site 2 was detected in the precipitates by PCR. As a positive control, PCR was also performed on 2% of the ChIP input. Gel is a representative experiment from 3 replicates. Diagram represents the amplicon region used for Site 1 and Site 2 detection. (B) ChIP-qPCR using SimpleChIP Enzymatic Chromatin IP Kit (Cell Signaling) was performed on fibroblasts infected for 48 h and pulsed with EGF for 1h, fibroblasts expressing EGR1_3xFlag_ infected for 48 h, or pure population of infect CD34^+^ HPCs in long-term culture for 5 days (6 dpi total). Fibroblasts were infected at an MOI of 1 and the CD34^+^ HPCs were infected at an MOI of 2. The presence of EGR1 Site 1 or Site 2 sequence was quantified by qPCR relative to a 2% input control and normalized to WT levels.

To confirm binding of EGR1 to putative binding sites in the UL133-UL138 locus, we transduced fibroblasts with lentiviruses expressing EGR1 fused to a 3xFlag epitope tag (EGR1_3xFlag_) and infected the cells with either a wild-type or a UL133-UL138-deletion mutant (negative control; NC) viruses. At 48 hpi, chromatin was crosslinked, isolated and digested for chromatin immunoprecipitation (SimpleChIP, Cell Signaling Technologies). Fragment sizes were confirmed to be 150-300bp in size using a bioanalyzer. Chromatin was immunoprecipitated with antibodies to either EGR1, Histone 3 (H3) or normal IgG and binding was detected by PCR with site-specific primers (Fig 5A). The EGR1 antibody precipitated sequences in the wild-type infection for both binding sites by PCR; but not in the NC or IgG control. These results indicate that EGR1 interacts with both site 1 and site 2.

We next analyzed EGR1 binding to UL133-UL138 sequences in infected fibroblasts (2 dpi) stimulated with EGF or overexpressing a Flag epitope-tagged EGR1 (EGR1_3xFlag_) and in CD34+ HPCs (6dpi) by ChIP combined with qPCR (Fig 5B). Site 1 and Site 2 sequences were increased 10- and 100-fold in the EGR1 pull down from fibroblasts where EGR1 was induced by EGF or overexpressed relative to the IgG control, respectively. Strikingly, in infected CD34^+^ HPCs EGR1 binding at site 1 and 2 was 1000-fold greater relative to the IgG control. These data indicate that EGR1 binds to sequences upstream of UL138 in the contexts of infection in fibroblasts and CD34+ HPCs.

### EGR1 induces UL138 protein accumulation

*UL138* is expressed from a series of overlapping 3’ co-terminal transcripts, many initiating downstream of the UL135 ORF [26, 42, 43]. The presence of putative EGR1 binding sites upstream of these transcripts suggests the existence of a promoter element in this region to regulate *UL138* expression; this element has yet to be mapped. In this case, EGR1 binding would be expected to induce *UL138*, but not *UL135* gene expression, consistent with our result in Fig 4A.

**Fig 6:**
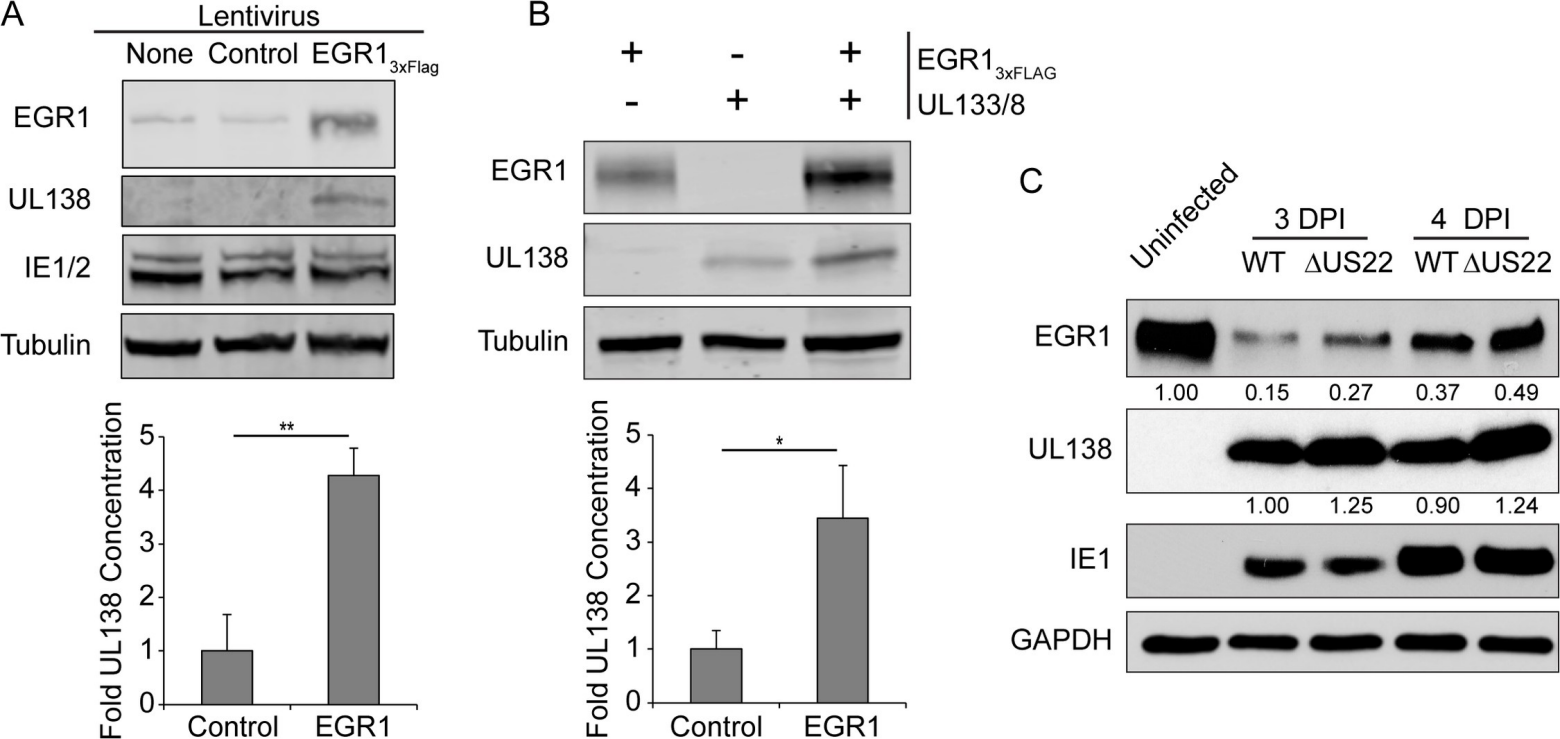
EGR1 promotes UL138 protein expression. (A) Fibroblasts were transduced with either EGR1_3xFlag_ or empty vector control and infected with WT TB40/E_GFP_ (MOI = 1) 24 hours later. At 48 hpi, protein lysates were separated by SDS-PAGE and proteins detected using α-FLAG, α-*UL138*, and α-Tubulin. (B) HEK293T cells were co-transfected with EGR1_3xFlag_, *UL133/8* encoding plasmid, or empty vector controls (minus sign). Protein lysates were harvested at 48h, samples were separated by SDS-PAGE and proteins detected using α-FLAG, α-*UL138*, and α-Tubulin. (A-B) *UL138* protein levels from either 4(A) or 3(B) independent experiments were normalized to the control and shown in graphs. Statistical significance was calculated by student t-test; asterisks indicate * p-value < 0.05 and ** p-value < 0.01. Error bars represent SEM. (C) Fibroblasts were infected with 1 MOI of WT or ΔmiR-US22 TB40/E_GFP_ virus and serum starved overnight before treating with 50 ng/mL of EGF. Samples were collected at 3 and 4 dpi, then separated on a SDS-PAGE gel, and blotted for α-EGR1, α-*UL138*, α-IE1, and α-GAPDH. Normalized values for EGR1 and *UL138* protein are below each band. Blot is representative of two independent experiments.

To determine if EGR1 is sufficient to induce *UL138* expression we transduced fibroblasts with lentivirus expressing either EGR1_3xFlag_ or empty vector and infected cells with TB40/E. EGR1 overexpression increased *UL138* expression in infected cells by 4-fold, while IE1 and 2 protein levels were unaffected (Fig 6A). To ensure that EGR1 activity is not priming UL138 expression from a more distal promoter site, we co-transfected HEK-293T cells with a plasmid containing the entire *UL133-UL138* locus in a promoterless vector backbone (UL133/8) and either the EGR1_3xFlag_ expression construct or an empty control. We detected a 3.5-fold increase in UL138 protein in the context of EGR1 overexpression relative to the empty vector control (Fig 6B). These results indicate that EGR1 expression is sufficient to stimulate *UL138* expression from an unmapped regulatory element encoded with the *UL133/8* locus.

Similar to other herpesviruses, CMV encodes a number of microRNAs [44, 45]. CMV miR-US22 targets EGR1 in the context of infection in CD34+ HPCs [46]. miR-US22 expression decreases EGR1 protein levels by 2- to 5- fold, reducing hematopoietic cell proliferation, the potential for multipotent hematopoietic colony formation and the frequency of CMV reactivation. These results emphasize the importance of EGR1 for hematopoietic differentiation and CMV reactivation. If EGR1 regulates *UL138* gene expression, then a miR-US22-mutant (ΔUS22) virus, which has increased expression of EGR1 and fails to reactivate, would be expected to also have increased UL138 protein. To test this, we infected fibroblasts with a WT or ΔUS22-mutant virus and pulsed them with EGF for 1 hour at different time points. At both 3 dpi and 4 dpi, UL138 was increased by ~25% in the ΔUS22-mutant virus infection relative to WT infection (Fig 6C). The increase in UL138 protein levels corresponded to increased EGR1 protein levels in the context of ΔUS22 virus infection. This result is consistent with the regulation of UL138 by EGR1 and suggests an epistatic relationship between UL138 and miR-US22 and EGR1 in regulating infection.

### CMV maintains EGR1 levels during latent infection but reduces its expression during productive replication

**Fig 7:**
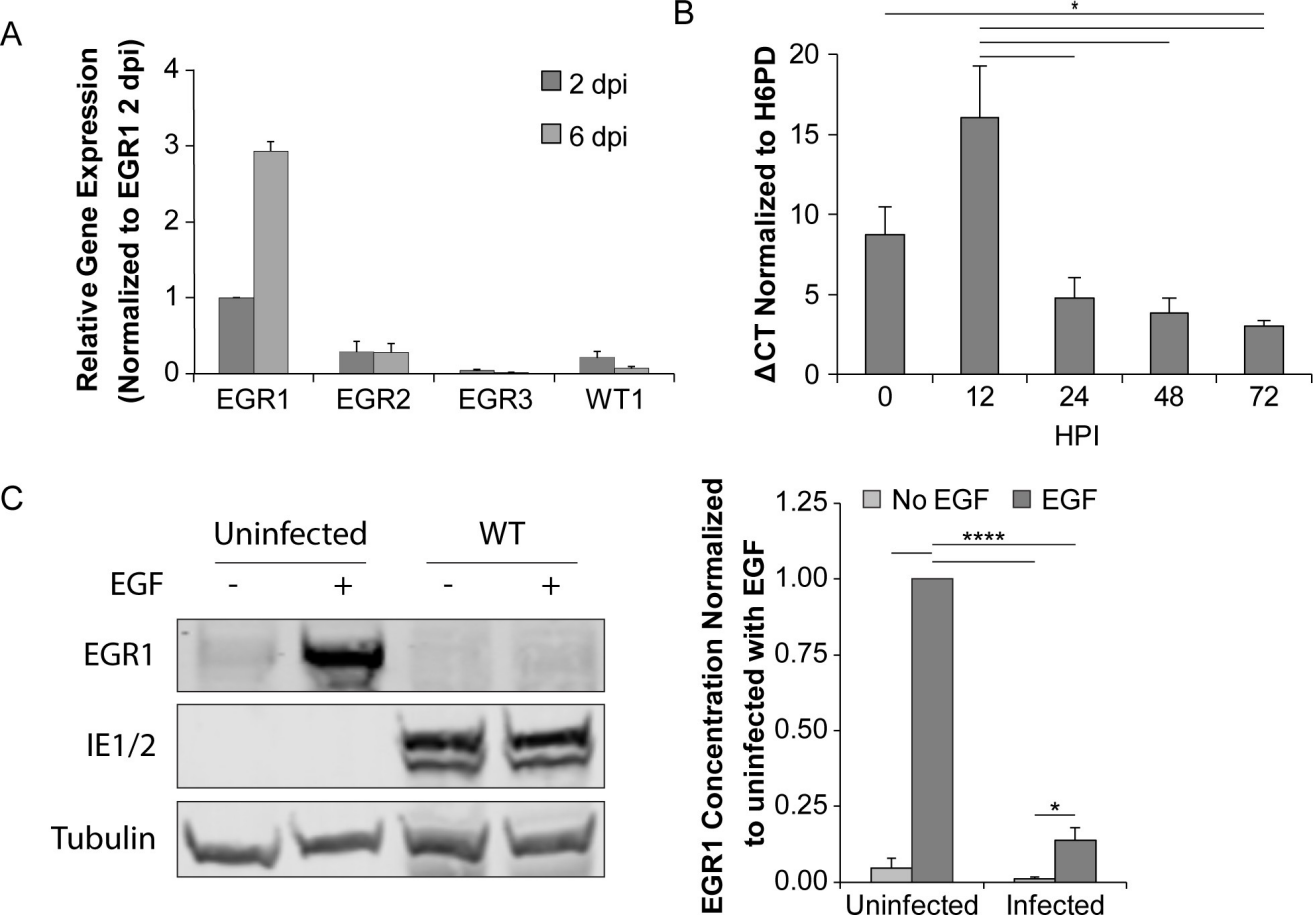
EGR1 levels are elevated during latent infection in CD34^+^ HPCs. (A) CD34^+^ HPCs were infected with TB40/E_GFP_ (MOI = 2). At 2 and 6 dpi, mRNA libraries were prepared for Illumina sequencing. Relative expression of EGR1, EGR2, EGR3, and WT1 was calculated by fragments per kilobase per million reads (FPKM) and normalized to EGR1 2 dpi levels. Error bars represent the range of gene expression between two independent donors. (B) Fibroblasts were infected with WT TB40/E_GFP_ (MOI = 1) and RNA was isolated at 0–72 hpi. EGR1 mRNA was quantified relative to H6PD by RT-qPCR. Results from 3 independent replicates are graphed error bars represent SEM. Statistical significance was calculated by One-Way ANOVA with Tukey’s correction and represented by an asterisk (* p-value < 0.05). (C) Fibroblasts were infected with TB40/E_GFP_ (MOI = 1) and transferred to serum-free media at 24 hpi. At 48 hpi, samples were pulsed with 10 nM of EGF for 1 h. Lysates were separated by SDS-PAGE and immunoblotted with α-EGR1, α-IE1/2, and α-tubulin. EGR1 protein levels were normalized to the uninfected sample stimulated with EGF and the mean from 3 independent replicates is graphed. Error bars represent SEM. We calculated statistical significance by two-way ANOVA with Tukey’s correction and represented significance by asterisks (* p-value < 0.05; **** p-value < 0.0001).

To determine how EGR1 is regulated during CMV latent infection, we analyzed EGR1 mRNA expression in TB40/E-infected CD34^+^ HPCs derived from two donors at 2 and 6 dpi by RNA sequencing [2]. In each donor, EGR1 expression increased following CMV infection from 2 to 6 dpi by 3-fold (Fig 7A). By contrast, the expression of two related genes belonging to the same zinc-finger transcription factor family, EGR2 and EGR3, were unchanged by CMV infection. Additionally, CMV did not affect expression of Wilms tumor 1 (WT1) a factor that binds competitively to the EGR1 consensus sequence to antagonize EGR1 transcriptional control of genes, including EGFR [47, 48]. By contrast, EGR1 expression is reduced 3-fold during replication in fibroblasts following an initial induction (Fig 7B) that is likely due to the stimulation of EGFR at the cell surface during viral entry [16, 49]. Further, EGR1 is strongly induced in serum starved, uninfected fibroblasts and infection diminishes this induction by 7-fold (Fig 7C). The reduced responsiveness of EGR1 to EGF stimulation in infected cells likely reflects diminished EGFR levels and signaling in the context of infection beginning at 24 hpi (Figs 1 and 2). These findings are consistent with the differential regulation of EGFR during productive and latent states of viral infection which we previously described [15].

### Mutation of EGR1 binding sites in the UL133-UL138 region ablates UL138 protein accumulation

**Fig 8:**
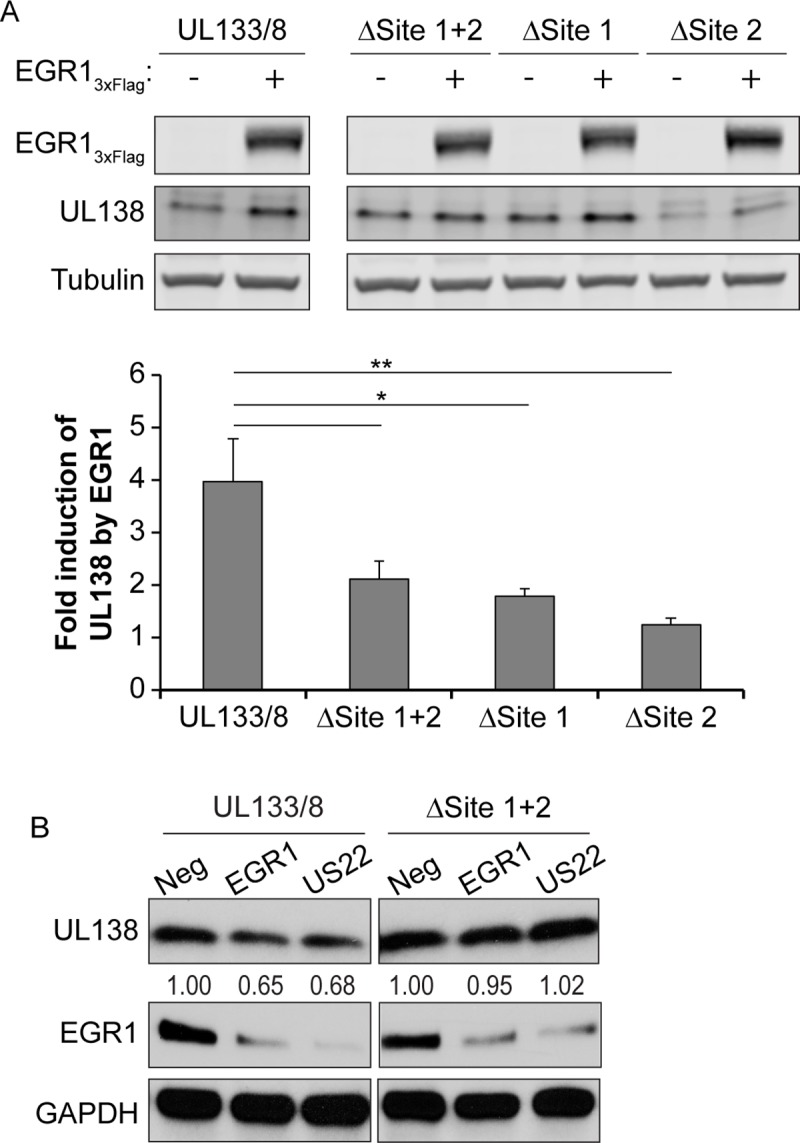
Mutation of EGR1 binding sites blocks induction of *UL138*. (A) HEK293T cells were co-transfected with either empty vector (minus sign) or EGR1_3xFLAG_ and a promoterless plasmid containing UL133/8 sequences or UL133/8 where EGR1 sites were mutated in combination (ΔSite1+2) or individually (ΔSite 1 or ΔSite 2). At 48 h, lysates were separated by SDS-PAGE, and proteins detected using α-Flag, α-UL138, and α-tubulin. *UL138* protein levels in EGR1_3xFLAG_ transfections were normalized to control levels to determine *UL138* induction. The results from 4 independent replicates are graphed. Statistical significance was calculated by One-Way ANOVA with Bonferroni correction (* p-value < 0.05 and ** p-value < 0.01). (B) HEK293T cells were co-transfected with the UL133/8 vector or the UL133/8 vector where EGR1 sites (ΔSite1, ΔSite 2) were disrupted and negative control siRNA, EGR1 siRNA, or miR-US22. Cells were transferred to serum-free media at 24 h. At 48 h, samples were stimulated with 50 ng/mL EGF for 1h and then lysed, separated by SDS-PAGE, and proteins detected using α-UL138 and α-GAPDH. *UL138* levels are normalized to negative control. A representative blot of 2 independent experiments is shown.

To validate the EGR1 binding sites upstream of *UL138*, we introduced silent mutations into wobble positions of codons within site 1 and site 2 (ΔSite1+2) or each site individually (ΔSite 1 or ΔSite 2) by site directed mutagenesis in the promoterless *UL133/8* vector construct (S4 Fig). HEK-293T cells were co-transfected with an empty vector or EGR1_3xFLAG_ and either WT *UL133/8* or ΔSite 1+2, ΔSite 1 or ΔSite 2. UL138 protein levels in cells expressing EGR1_3xFlag_ were normalized to that in cells transfected with empty vector (Fig 8A). EGR1 overexpression induced UL138 protein accumulation 4-fold from the WT UL133/8 construct; however mutation of either Site 1 or Site 2 resulted in 2-fold reduced levels of UL138 protein accumulation.

If EGR1 regulates UL138 gene expression through binding to the sites upstream of UL138, then we hypothesized that EGR1 knockdown by siRNA or the CMV miR-US22 would decrease UL138 expression in EGR1 binding site-dependent manner. We analyzed UL138 protein levels in HEK 293T cells transfected with the WT UL133/8 or ΔSite1+2 promoterless vectors and EGR1 siRNAs or miR-US22 to knockdown EGR1 (Fig 8B). Knockdown of EGR1 either with an EGR1 siRNA or miR-US22 decreased UL138 protein levels by 30% in cells containing the wild-type *UL133/8* plasmid. However, EGR1 knockdown had no effect on UL138 protein accumulation in cells where site 1 and site 2 were disrupted. Taken together, these results further validate the significance of the EGR1 binding sites in the viral genome to the regulation of *UL138* expression.

**Fig 9:**
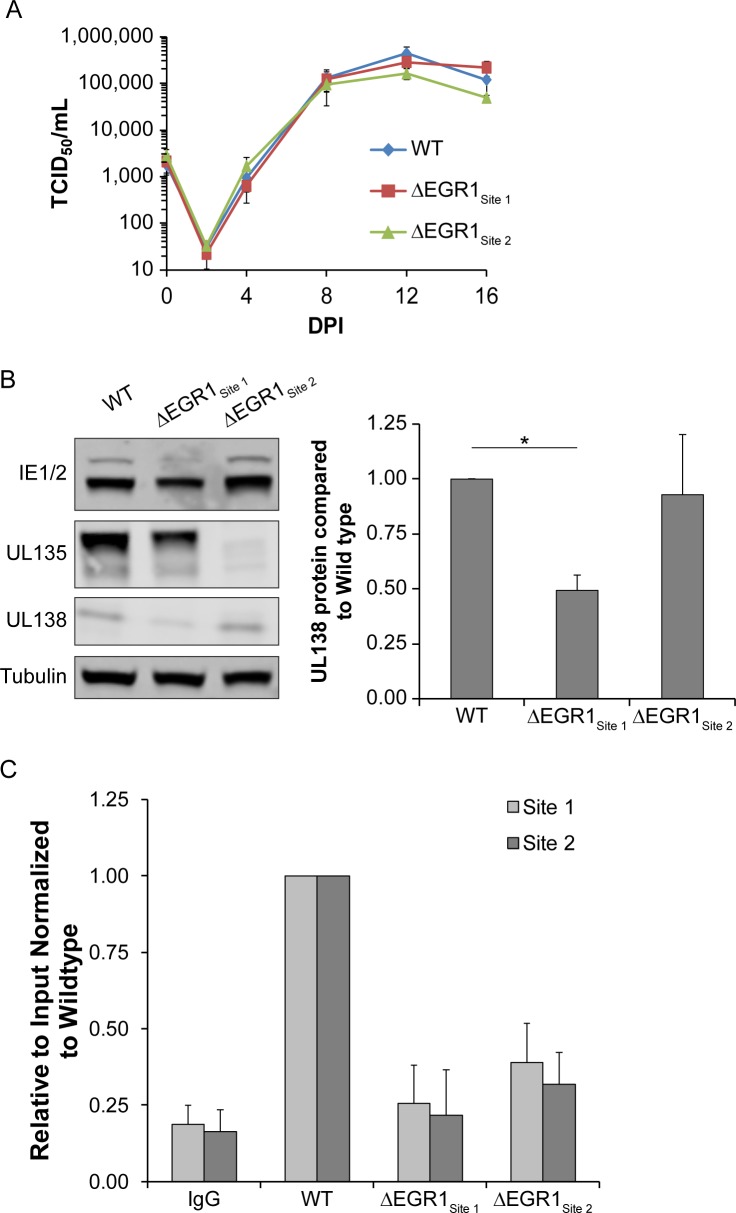
Disruption of either EGR1 site blocks EGR1 binding during infection. (A) Fibroblasts were infected with WT TB40/E_GFP_ or EGR1 binding mutant viruses, ΔSite 1 or ΔSite 2 (MOI = 0.02). Cells and media were collected from 0–16 dpi and virus titers measured by TCID_50_. The average of 3 independent replicate experiments is shown. (B) Fibroblasts were infected with WT or EGR1 binding mutant viruses. Samples were lysed at 48 hpi, separated by SDS-PAGE and proteins detected using α-IE1/2, α-UL135, and α-UL138, and α-tubulin. UL138 protein levels were quantified and each mutant was normalized to WT over 3 independent experiments. The average value is graphed with error bars representing SEM and statistical significance is calculated by One-way ANOVA with Bonferroni correction (* p-value <0.05). (C) Fibroblasts were infected with WT TB40/E_GFP_ or EGR1 binding mutant viruses, ΔSite 1 or ΔSite 2, (MOI = 1) and transferred to serum-free media at 24 hpi. At 48 hpi, samples were pulsed with 10 nM EGF for 1h and processed for ChIP-qPCR using SimpleChIP Enzymatic Chromatin IP Kit (Cell Signaling). The presence of EGR1 Site 1 sequence was calculated relative to a 2% input control and normalized to WT levels.

We next engineered the EGR1 binding site disruptions into the TB40/E genome cloned as a bacterial artificial chromosome, resulting in TB40/E-ΔEGR1_Site 1_ and TB40/E-ΔEGR1_Site 2_. We confirmed the disruption of EGR1 binding sites by sequencing. These viruses exhibited no defects for replication relative to a parental WT virus in fibroblasts when analyzed for multi-step growth (Fig 9A), indicating that the mutations introduced to disrupt EGR1 binding to the *UL133-UL138* region do not affect productive virus replication in fibroblasts.

To determine if either EGR1 binding site mutation affected *UL138* expression in the context of infection, we infected fibroblasts with TB40/E-WT, -ΔEGR1_Site 1_ or -ΔEGR1_Site 2_, and measured UL138, UL135, and IE1/IE2 protein levels at 48 hpi by immunoblot (Fig 9B). Disruption of Site 1, but not Site 2, decreased *UL138* protein levels by 50%. While, IE protein levels were unaffected, we detected a striking loss of UL135 protein in the TB40/E-ΔEGR1_Site 2_ infection. Several independent clones of this mutant produced the same phenotype.

To confirm the loss of EGR1 binding to ΔEGR1_Site 1_ in the context of infection, we transduced fibroblasts with EGR1_3xFLAG_ lentivirus and infected with TB40/E-WT, ΔEGR1_Site 1_ or ΔEGR1_Site2_ viruses. Chromatin was precipitated with antibodies to EGR1 (SimpleChIP, Cell Signaling Technologies) and binding to sites was quantified by qPCR using primers flanking site 1 or site 2, as was used if Fig 5. In two independent experiments, EGR1 precipitated 4-fold more site 1 and 2 sequence in the WT-virus infection relative to the IgG control (Fig 9C). Infection with TB40/E-Δ EGR1_Site 1_ or -ΔEGR1_Site2_ resulted in a 3- or 4-fold reduction, respectively, in EGR1 binding to site 1 or 2 relative to WT. Taken together, these results demonstrate that EGR1 binds to its sites in UL133-UL138 and that EGR1 binding induces *UL138* expression.

### EGR1-stimulation of UL138 is required for CMV latency

**Fig 10:**
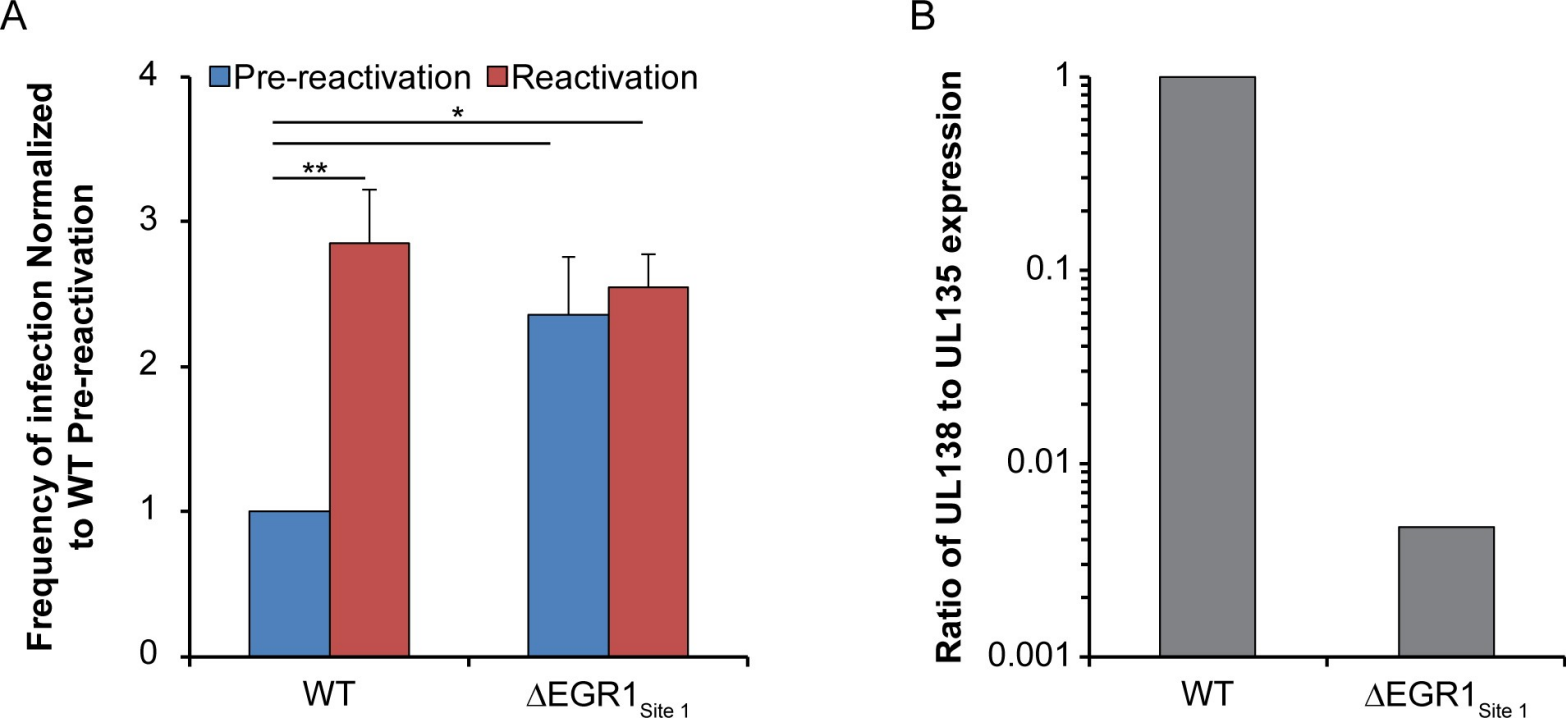
Inhibition of EGR1 binding to site 1 disrupts CMV latency. (A) CD34^+^ HPCs were infected with either WT TB40/E_GFP_ or TB40/E-ΔEGR1_Site 1_ mutant virus (MOI = 2). At 24 hpi, CD34^+^/GFP^+^ cells were sorted and seeded into long-term culture. After 10 days in culture, parallel populations of either mechanically lysed cells or whole cells were plated onto fibroblast monolayers in cytokine-rich media. 14 days later, GFP+ wells were scored and frequency of infectious centers was determined by extreme limited dilution analysis (reactivation). The mechanically lysed population defines the quantity of virus present prior to reactivation (pre-reactivation). The frequency was normalized to WT pre-reactivation and the average of three independent experiments is shown. Statistical significance was calculated by Two-Way ANOVA with Tukey’s correction and represented by asterisks (* p-value < 0.05). (B) At 5 dpi, RNA was isolated from WT- and ΔEGR1_Site1_-infected CD34^+^ HPCS and transcripts encoding *UL135* and *UL138* was quantified by RT-qPCR. A ratio of UL138 to UL135 contain transcripts was calculated with Pfaffl.

To determine if EGR1 binding is important for CMV latency we infected CD34^+^ HPCs with either TB40/E-WT or TB40/E-ΔEGR1_Site 1_ mutant virus. Given that UL135 expression is disrupted in multiple independent clones of ΔEGR1_Site2_ and the importance of UL135 for reactivation [17], we excluded this virus from further analysis. At 24 hpi, pure populations of infected HPCs (GFP^+^/CD34^+^) were isolated and seeded into transwells above a stromal cell support for long-term culture. As described for Fig 3B, live cells or a cell-equivalent lystate control was seeded onto fibroblast monolayers by limiting dilution. GFP^+^ wells were scored 14 days later to determine the frequency of infectious centers present at the time of lysis (pre-reactivation) or resulting from reactivation (live cells). Reactivation of TB40/E-WT produced a 3-fold increase in the frequency of infectious centers relative to the pre-reactivation control (Fig 10A). In contrast, TB40/E-ΔEGR1_Site 1_ infection resulted in a loss of latency and equal frequencies of infectious centers were measured prior to and following reactivation. The loss of latency with the ΔEGR1_Site 1_ mutant is a similar phenotype as a *UL138*_*null*_-mutant virus in CD34^+^ HPCs [17]. To determine if expression of *UL138* was diminished in the absence of EGR1 binding in CD34^+^ cells, we analyzed UL138 transcripts at 5 dpi by RT-qPCR in cells infected with WT or ΔEGR1_Site 1_. Because UL138 is encoded on a series of 3’ co-terminal polycistronic transcripts that differ in their 5’ ends (some that extend 5’ of the EGR1 binding site), we determined the ratio of UL138 transcripts to larger transcripts that include UL135 sequences (upstream of Site1). The UL138-to-UL135 ratio of transcripts was 215-fold greater in the WT compared to ΔEGR1_Site1_ infection (Fig 10B). These results are consistent with the requirement for UL138 in latency and demonstrate a role for EGR1 in driving UL138 gene expression for latency.

## Discussion

All herpesviruses rely on and manipulate cellular signaling pathways to regulate their viral lifecycle—and this is particularly important to the establishment and maintenance of latency and reactivation from latency. CMV manipulates EGFR and its downstream pathways to achieve this goal [11, 15, 18, 28, 30]. EGFR is downregulated during the replicative cycle and further inhibition enhances viral replication [15, 27, 28]. Stimulation of EGFR signaling upon entry into hematopoietic cells is important to establish an environment to support latency in CD34+ HPCs [10, 16, 50]. Sustained EGFR signaling is important for latency and inhibition of EGFR or its downstream pathways is important for viral reactivation [15]. Targeting EGFR at entry, replication, latency and reactivation provides CMV with access to cellular pathways involved in survival, differentiation, proliferation, motility, immune signaling, and DNA repair [51–55]. While the precise downstream effects of virus-mediated control of EGFR that are important to CMV infection are not clearly defined, here we demonstrate that signaling downstream of EGFR impacts expression of the latency determinant UL138 from the viral genome and that this regulation of UL138 gene expression is important to the latent infection.

**Fig 11:**
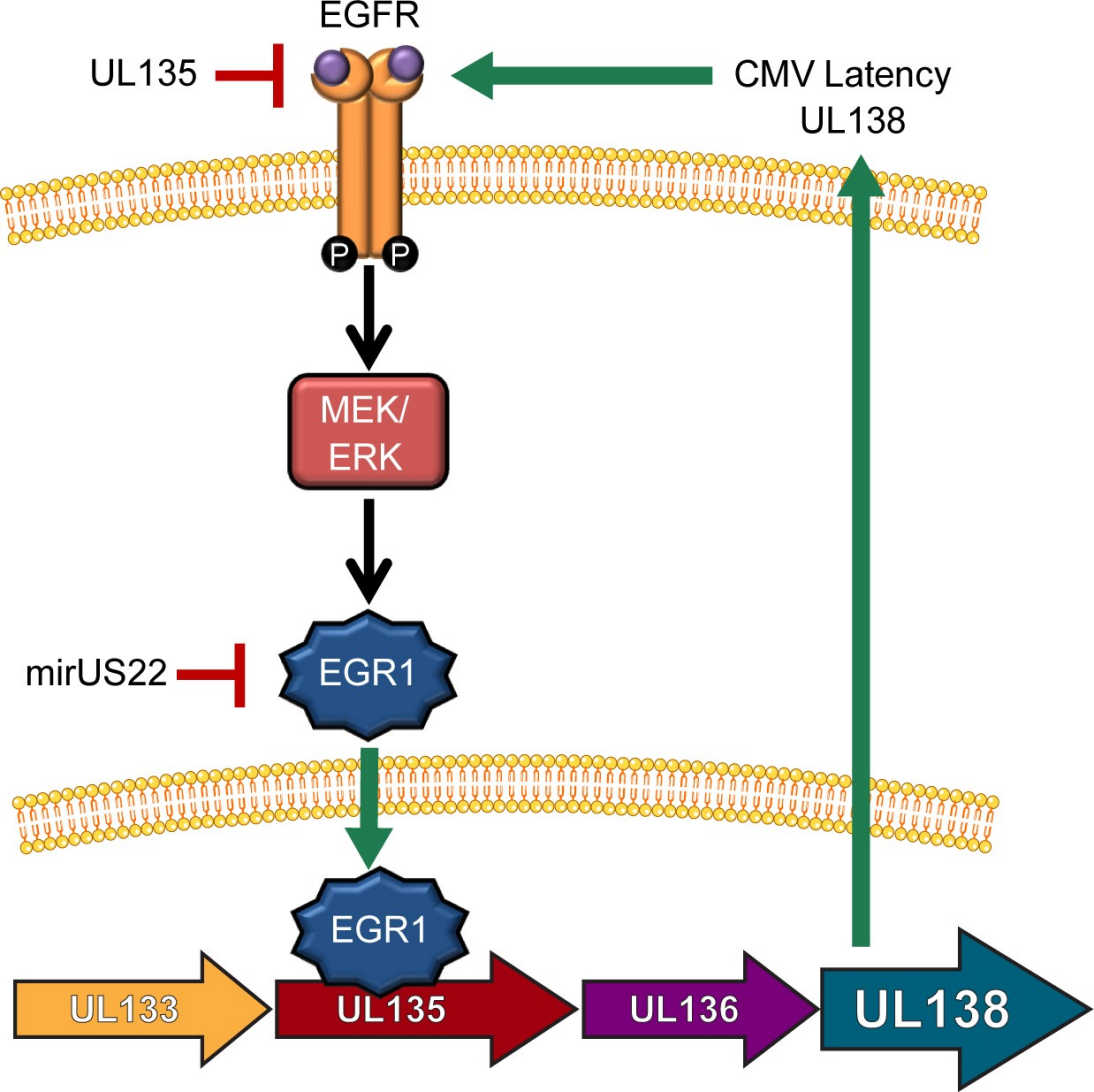
Model for the regulation of UL138 expression through EGFR induction of EGR-1 in a positive feedback mechanism. Our data demonstrates that EGFR signaling promotes EGR-1 expression through MEK/ERK signaling pathways. EGR-1 stimulates UL138 expression and UL138 feeds back to sustain EGFR signaling. UL135 targets EGFR for turnover and CMV miR-US22 targets EGR1 to negatively regulate this cycle for reactivation.

The EGR1 transcription factor is induced by the MEK/ERK and PI3K/AKT pathways downstream of EGFR. EGR1 plays a critical role in hematopoietic differentiation, in maintaining stemness in hematopoietic stem cells [19] and later in macrophage differentiation [20, 21, 56]. Intriguingly, EGR1 restricts hematopoietic differentiation along the macrophage lineage at the expense of granulocyte and erythroid lineages [21, 56]. Here we show that EGR1 binds upstream of UL138 and induces the accumulation of UL138 (Figs 5, 6, 8 and 10B). EGR1 stimulation of UL138 is inducible through the activation of EGFR (Figs 5B and 7C) and diminished if the downstream MEK/ERK pathway, but not PI3K/AKT, is inhibited (Fig 4D). Viruses where EGR1 binding sites have been ablated, express lower levels of UL138 and fail to establish latency (Figs 8A, 9B and 10). Finally, CMV miR-US22 knockdown of EGR1 is required for reactivation [46] and reduces UL138 protein levels (Figs 6C and 8B). From this work, a model emerges whereby high levels of EGR1 in CD34^+^ HPCs [19] primes these cells for expression of *UL138* and the establishment of latency upon infection (Fig 11). Combined with our previous findings that *UL138* maintains EGFR signaling [15], we propose a model by which EGFR signaling through MEK/ERK induces EGR1 to drive *UL138* expression. UL138 feeds back to sustain EGFR signaling to promote CMV latency. Disrupting this feedback loop, with either chemical inhibitors for EGFR or downstream MEK/ERK pathways or by preventing EGR1 binding, results in increased replication in CD34+ HPCs. To ensure reactivation the virus encodes both UL135 [15, 17, 18] and miR-US22 [46], which target EGFR and EGR1, respectively.

During a productive infection, we demonstrate a striking and early downregulation of EGFR from the cell surface and a decrease in total levels (Fig 1). The reduction in EGFR corresponds to a loss of responsiveness in major pathways downstream of EGFR (Fig 2 and S1 Fig). Downregulation of PI3K and AKT pathways is most important for productive replication, whereas inhibition of STAT signaling negatively impacted replication in fibroblasts (Fig 3A). By contrast, in the context of latency CD34+ cells, inhibition of MEK/ERK, STAT or PI3K/AKT pathways resulted in enhanced reactivation (Fig 3B), as is also observed using EGFR inhibitors [15]. MEK/ERK signaling is particularly interesting since EGR1 was induced through MEK/ERK (and not PI3K/AKT) in CD34+ HPCs (Fig 4D). As inhibition of PI3K or AKT strongly stimulates reactivation from latency [15] (Fig 3B), this indicates that the PI3K/AKT pathways contribute to latency through a mechanism that is distinct from the regulation of EGR1 and UL138 gene expression. This will be an important area of inquiry for future studies.

The interplay between PI3K/AKT and MEK/ERK pathways in the regulation of replication and latency is evident in the context of other herpesvirus infections. Herpes simplex virus 1 (HSV-1) activates PI3K activity through stimulation of neural growth factor to maintain latency [57]. Inhibition of P13K stimulates HSV-1 reactivation, but full reactivation also requires c-Jun N-terminal kinase (JNK), a MAPK family member, signaling to induce histone phosphorylation on viral promoters to de-repress HSV-1 gene expression [58]. Also, HSV-1 proteins VP11/12 interact with Src-family kinases, Grb2, Shc, and p85 through a tyrosine-binding motif in order to stimulate PI3K/AKT activity without growth factor stimulation [59]. HSV-1 US3 protein kinase suppresses ERK signaling to promote viral replication [60]. Epstein-Barr virus (EBV) latency membrane protein LMP-1 promotes both EGFR protein levels and activation of STAT3 and ERK signaling pathways [61–63], while LMP2A activates PI3K/AKT signaling [64, 65]. Additionally, LMP2A also promotes cellular survival through ERK activation mediating proteosomal degradation of proanoikis mediator Bim [66]. Lastly, Kaposi’s sarcoma-associated herpesvirus (KSHV) latent infection promotes PI3K activation [67]. However, KSHV activates MEK/ERK signaling to promote its reactivation and inhibition of MEK/ERK signaling suppresses ORF50 expression and KSHV reactivation [68, 69]. These combined findings illustrate the significance of PI3K/AKT and MEK/ERK signaling pathways to the regulation of herpesvirus programs of latency and replication.

The role of signaling pathways in CMV infection, particularly, MEK/ERK is complex. MEK/ERK is an activator of mitogen activated protein kinase (MAPK) and has previously been shown to be important for reactivation from latency in CD34+ progenitor, CD14+ monocyte, and dendritic models of latency [70–74]. In those studies, ERK signaling upregulated the pro-survival Mcl-1 host factor and inhibition of ERK results in increased cell death in the THP-1 monocytic cell line or CD34+ HPCs [71]. Further, ERK signaling was also shown to upregulate ELK-1 in CD34+ cells to counter the stimulation of pro-apoptotic pathways [72]. By contrast, in our study inhibition of ERK did not result in any detectable increase in cell death relative to the DMSO control or other inhibitor treatments (S3 Fig). The MAPK pathway was shown to be attenuated by CMV US28 for latency in monocytes and inhibition of MAPK or NFκB pathways reduced replication [73]. However, another study showed that while US28 ligand-dependent signaling is important to latency, US28 plays an important role in reactivation [75]. The differences in these studies reflects the complexity of signaling in hematopoietic cells, which is likely magnified by differences hematopoietic cell type or subpopulation composition across primary cell model systems and differences inherent to experimental systems.

The role of EGR1 in promoting *UL138* expression during CMV infection is particularly intriguing because CD34^+^ HPCs express high levels of EGR1 in the bone marrow [19]. As such, CD34^+^ cells are predisposed to drive *UL138* expression upon CMV infection to suppress viral replication. This observation is one possible explanation for why we detect the UL138 protein, but not other UL133-UL138 proteins, in latently infected CD34^+^ HPCs [26] and may underlie differential expression of UL133-UL138 genes in different cell types. Upon differentiation of CD34^+^ HPCs EGR1 expression falls rapidly [19], a requirement for the differentiation and migration of stem cells out of the bone marrow. This diminishment EGR1 would be expected to result in decreased UL138 expression, predisposing cells towards reactivation. By contrast, EGR1 levels are low in sites of productive replication, such as fibroblasts. CMV-mediated suppression of EGFR and its downstream signaling further suppress EGR1 and consequently UL138 expression in contexts of replication (Figs 2 and 7C). Finally, CMV replication in fibroblasts stimulates WT1 expression [28], which competes antagonistically for EGR1 targets, including EGFR [47, 76], and indicates another mechanism by which the virus antagonizes EGFR/EGR1 signaling for productive infection. Finally, the Nelson lab demonstrates that CMV miR-US22 targets EGR1 to promote hematopoietic differentiation and reactivation, and results in decreased UL138 [46] (Figs 6C and 8B). Taken together, our results demonstrate that EGR1 is an important target in CMV infection and its expression is targeted through multiple mechanisms to promote viral replication.

EGR1 regulates viral gene expression in the context of other herpesvirus infections. In HSV-1, EGR1 binding sites are located within the introns of both ICP22 and ICP4 [77]. In contrast to our findings, EGR1 inhibits both ICP4 and ICP22 by blocking SP1 binding sites and recruiting the co-repressor Nab2 [77]. The authors predict that the inhibition of both of these immediate early genes would promote HSV-1 gene silencing for the establishment of latency. EBV transactivator ZTA upregulates EGR1 by both interacting with its promoter and by increasing ERK signaling in order to promote viral reactivation[78]. While the mechanism by which each herpesvirus utilizes EGR1 to control viral latency and replication differs, it is clear that manipulating EGR1 is common feature. These differences may reflect the unique cell types where each herpesvirus family member establishes latency.

Herpesviruses manipulate multiple signaling pathways to control viral latency and to promote viral replication, and understanding the complex interplay between these signaling pathways and the virus is necessary to fully appreciate how these viruses persist. We have shown that viral manipulation of host signaling impacts the control of *UL138* expression. Putative EGR1 binding sites exist throughout the CMV genome and, because EGR1 can either promote or repress gene expression [76, 79], it will be important to understand how EGR1 binding to promoters across the genome impact latent and replicative states of infection.

## Materials and methods

### Cells

MRC-5 lung fibroblasts (ATCC), HEK293T/17 cells (ATCC), Sl/Sl stromal cells (Stem Cell Technology), M2-10B4 stromal cells (Stem Cell Technology), and CD34^+^ HPCs were maintained as previously described [17]. Human CD34^+^ HPCs were isolated from de-identified medical waste following bone marrow isolations from healthy donors for clinical procedures at the Banner-University Medical Center at the University of Arizona. Latency assays were performed as previously described [15, 17].

### Viruses

Bacterial artificial chromosome (BAC) stocks of TB40/E WT virus, a gift from Christian Sinzger [80], was engineered to express GFP from a SV40-promoter [26]. EGR1 binding mutant viruses were created by two-step, positive-negative selection approach with galK substitution as was previously described [17, 81]. Both the TB40/E *UL133/8*_Null_ galK intermediate and pGEM-T *UL133-UL138* shuttle vector, referred to as *UL133/8* plasmid in the results, were created previously and described in Umashankar et al. 2014 [17]. Creation of TB40/E mutant lacking the *UL133-138* locus is described in Umashankar et al. 2011 [26]. EGR1 binding sites were mutated by Phusion PCR mutagenesis using flanking PCR primers to each region with mutations incorporated into the corresponding forward and reverse primers (Table 1). pGEM-T *UL133-UL138* plasmids removing EGR1 site 1 (ΔSite 1), EGR1 site 2 (ΔSite 2), or both EGR1 sites (ΔSite 1+2) were created and stocks were maintained in DH10B bacteria glycerol stocks. Inserts for BAC recombineering were gel purified from pGEM-T ΔSite 1 and ΔSite 2 plasmids digested with EcoRI. Inserts were electroporated into SW102 *E*. *coli* containing the TB40/E UL133/8_Null_ galK intermediate as previously described [82]. BAC integrity was confirmed by comparing EcoRV digestion of the EGR1 binding site mutant BACs to WT TB40/E BAC digest. Mutations of EGR1 binding sites in TB40/E-ΔEGR1_Site 1_ and TB40/E-ΔEGR1_Site 2_ were confirmed by Sanger sequencing. Loss of EGR1 binding in TB40/E-ΔEGR1_Site 1_ was confirmed by ChIP-qPCR, described below. TB40/E_GFP_ΔmiR-US22 was created as described in Mikell et al. [46].

**Table 1:** Primer sequences.

Primer	Sequence
ΔSite 1 Forward	5’- [phos]CCAACCCCGCAGGTGCCGCG​-3’
ΔSite 1 Reverse	5’- [[phos]GGGGTGGGTGGCCACC-3’
ΔSite 2 Forward	5’- [phos]CACCCCGATGGTCGGACATCGAGG-3’
ΔSite 2 Reverse	5’- [phos]GGGGGGCTAACTCGGAAACCG-3’
pCIG3 EGR1 Forward	5’- ATCGATCGGAATTCCACCATGGCCGCGGCCAAGGCC-3’
pCIG3 EGR1 Reverse	5’- GCATGCATTTAATTAATCAGCAAATTTCAATTGTCCTGGGAGAAAAGGTTGC-3’
EGR1 3xFlag Forward	5’-[phos]AAGATCATGACATCGATTACAAGGATGACGATGACAAGTGATTAATTAAGGG GATCCGCCCCTCT-3’
EGR1 3xFlag Reverse	5’- [phos]TATAATCACCGTCATGGTCTTTGTAGTCGCCGCCGCCGCCGCAAATTTCAAT TGTCCTGGGAGA-3’
EGR1 RT-qPCR Forward	5’-AGCCCTACGAGCACCTGAC-3’
EGR1 RT-qPCR Reverse	5’-GGGCAGTCGAGTGGTTTG-3’
EGFR RT-qPCR Forward	5’-CATGTCGATCGACTTCCAGA-3’
EGR1 RT-qPCR Reverse	5’-GGGACAGCTTGGATCACACT-3’
H6PD RT-qPCR Forward	5’-GGACCATTACTTAGGCAAGCA-3’
H6PD RT-qPCR Forward	5’-CACGGTCTCTTTCATGATGATCT-3’
Site 1 ChIP Forward	5’- CGCCGACGGAGCCGA-3’
Site 1 ChIP Reverse	5’- TGCACCGCCTTTTCCAAGAGTTC-3’
Site 2 ChIP Forward	5’- AATCTCTCGAAGGTGGGACTCT-3’
Site 2 ChIP Reverse	5’- CTCGGAAACCGACACGATAGG-3’
UL135 RT-qPCR Forward	5’-GCGGTGTACGTCGCTCTAC-3’
UL135 RT-qPCR Reverse	5’-GGAAACTCGGGTTTATCTATCG-3’
UL138 RT-qPCR Forward	5’-TGAGATCTTGGTCCGTTGG-3’
UL138 RT-qPCR Reverse	5’-GTGTGTTATCCGCGACGAC-3’

### Plasmids and lentiviruses

pDONR221 containing EGR1 cDNA was purchased from DNASU (Arizona State University; Phoenix, Az). EGR1 was PCR amplified from the pDONR221 plasmid with pCIG3 EGR1 forward and reverse primes and inserted into pCIG3 plasmid at PacI and EcoRI digestion sites. Addition of a 3xFlag epitope tag was done by Phusion PCR mutagenesis EGR1 3xFlag Forward and Reverse primers EGR1_3xFlag_ Forward and Reverse sequences. All plasmid inserts were verified through Sanger sequencing and maintained in DH10B glycerol stocks. EGR1_3xFlag_ lentivirus was created by cotransfecting pCIG3 EGR1_3xFLAG_, pMD2.G, and psPAX2 (gifts from Didier Trono; Addgene #12259 and 12260; http://n2t.net/addgene:12259; RRID:Addgene_12259; http://n2t.net/addgene:12260; RRID:Addgene_12260) into HEK293T/17 cells with polyethylenimine (Polysciences) and collected supernatants at 48 and 72h post transfection. Plasmid transfections were carried out in HEK293T/17 cells using PEI at 1 μg of DNA to 3 μg PEI. Plasmid encoding shRNA of EGR1 was described in Mikell et al. [46].

### Flow cytometry

MRC-5 fibroblasts were infected with 1 MOI of TB40/E_GFP_ virus for 0–72 hpi. Cells were lifted off the plates, fixed in 2% Formaldehyde in PBS for 30 min, and washed with excess PBS. Cells were then stained with Brilliant Violent 421-conjugated α-EGFR (Biolegend; Table 2). Samples were gated for intact GFP^+^ cells and geometric mean of fluorescence intensity (geoMFI) was measured using a BD LSRII equipped with FACSDiva Software (BD Bioscience Immunocytometry Systems) and FlowJo software.

**Table 2:** Antibody description and sources.

Antibody	Species	Source	Concentration
AKT	rabbit	Cell Signaling	Western: 1:1000
Alexa Fluor 546 anti rabbit	goat	Molecular Probes	IF: 1:7,000
Alexa Fluor 647 anti mouse	goat	Molecular Probes	IF: 1:7,000
Brilliant Violet 421 EGFR	mouse	BioLegend	Flow 5 μL/ 1x10^6^ cells
Dylight 700 conjugated anti mouse	goat	Pierce	Western: 1:12,000
Dylight 800 conjugate anti rabbit	goat	Pierce	Western: 1:12000
EGFR (D38B1)	rabbit	Cell Signaling	Western 1:10,00; IF 1:50
EGR1 (44D5)	Rabbit	Cell Signaling	Western 1:1,000; ChIP 10 μL/ 4x10^6^ cells
EGR1	rabbit	Bethyl	Western 1:1,000
Flag (D655B)	rabbit	Cell Signaling	Western 1:1,000
GAPDH (6C5)	mouse	Abcam	Western 1:15,000
HRP anti-mouse	goat	Jackson ImmunoResearch	Western: 1:5000
HRP anti-rabbit	goat	Jackson ImmunoResearch	Western: 1:5000
IE1 (8B1.2)	mouse	Millepore Sigma	Western: 1:40,000
IE1/2 (3H4)	mouse	Tom Shenk; Princeton University	Western 1:1,000
IE2	mouse	Tom Shenk; Princeton University	IF 1:50
IgG, Normal	rabbit	Cell Signaling	ChIP 2 μg/ 4x10^6^ cells
Histone H3 (D2B12)	rabbit	Cell Signaling	ChIP 10 μL/ 4x10^6^ cells
MEK1/2	rabbit	Cell Signaling	Western: 1:1000
PE conjugated CD34	mouse	BD Biosciences	Flow 20μL/1x10^6^ cells
phospho-AKT (S473; D9E)	rabbit	Cell Signaling	Western 1:2000
Dylight 649 conjugated phospho-AKT (S473)	mouse	Rockland	Flow: 1 μL/ 1x10^5^ cells
phospho-EGFR (Tyr1068; D7A5)	rabbit	Cell Signaling	Western 1:1000
phospho-p44/42 MAPK (ERK1/2) (Thr202/Tyr204) (D13.14.4E)	rabbit	Cell Signaling	Western 1:2000
Alexa Fluor 350 conjugated phospho-EGFR(Tyr1068)	mouse	R&D	Flow: 1 μL/ 2x10^5^ cells
phospho-MEK1/ (S217/221; 41G9)	mouse	Cell Signaling	Western 1:2000
Alexa Fluor 647 conjugated phospho-MEK1/ (S217/221	rabbit	BD Phosflow	Flow: 1 μL/ 2x10^5^ cells
UL135	rabbit	Open Biosystems ^a^	Western 2 μg/mL
UL138	rabbit	Open Biosystems ^a^	Western 2 μg/mL
α-Tubulin (DM1A)	mouse	Sigma	Western 1:10000

^a^ Custom Ordered antibody

CD34^+^ HPCs were infected with 2 MOI of TB40/E/E_GFP_ virus for 48 h. Cells were fixed in 2% formaldehyde in PBS on ice for 30 min. Washed with excess PBS. Cells were permabilized with Perm/Wash Buffer (BD Biosciences) containing 3% goat and 3% human serum for 30 min at room temp. Cells were stained with conjugated α-CD34, pEGFR(Y1068), pAKT(S473), pMEK1/2(S217/221), and pERK1/2(T202/Y204) antibodies (Table 2). Tubes were washed with excess Perm/Wash buffer. Samples were stored in 2% formaldehyde in PBS until analyzed using BD LSRII. Samples were gated for CD34^+^ GFP^+^ and geometric mean of fluorescence intensity (geoMFI) for each phosphorylation marker was measured using a BD LSRII equipped with FACSDiva Software (BD Bioscience Immunocytometry Systems) and FlowJo software.

### Immunoblotting

Lysates were separated by electrophoresis on precast 12% Tris-Bis SDS-PAGE gel (Genscript) or 4–20% precast gels (BioRad). Gels were transferred onto Immobilon-P PVDF membrane (EMD Millipore). Antibodies were incubated in with blocking solution, either 5% milk in TBS-T or 5% BSA in TBS-T, as per antibody manufacturer specifications. After antibody staining, blots were incubated with fluorescent secondary antibodies and imaged and quantitated using a Li-Cor Odyssey CLx infrared scanner with Image Studio software. Antibodies and sources are defined in Table 2. US22 experiments were developed using chemiluminescence with film and quantified with Image J software.

### RT-qPCR

Cells were infected with 1 MOI of TB40/E_GFP_ and RNA was isolated using Quick-DNA/RNA miniprep kit (Zymo Research) from 0–72 hpi. RNA was reverse transcribed into cDNA using Transcriptor First Strand cDNA Synthesis Kit (Roche). cDNA for EGR1, EGFR, and H6PD was quantified using LightCycler SYBR Mix kit (Roche) and corresponding primers (Table 1). Assays performed on Light Cycler 480 and corresponding software. ΔCT for EGR1 and EGFR were calculated by Pfaffl method normalized to H6PD [83].

For measuring EGR1 transcripts during chemical inhibition in CD34^+^ HPCs, cells were infected with 2 MOI of TB40/E_GFP_. At 4 hpi, cells were collected and divided equally for treatment with MEK/ERK and PI3K/AKT (see Table 3). After 48h, RNA was isolated using Quick-DNA/RNA miniprep kit (Zymo Research). RNA was reverse transcribed into cDNA and quantified for EGR1 and H6PD (Table 1), as described above. Relative expression for EGR1 was normalized to H6PD and compared to untreated samples using the Pfaffl method ($Relative\;expression=\frac{{{Eff}_{EGR1}}^{\left({CT}_{Untreated\;EGR1}-{CT}_{Treatment\;EGR1}\right)}}{{{Eff}_{H6PD}}^{\left({CT}_{Untreated\;H6PD}-{CT}_{Treatment\;H6PD}\right)}}$) [83].

**Table 3:** Chemical inhibitors.

Inhibitor	Target	Source	Concentration
Binimetinib	MEK1/2	LC Laboratories	1 μM
SCH772984	ERK1/2	Selleckchem	125 nM
Fludarabine	STAT1	Selleckchem	50 μM
S3I-201	STAT3	Selleckchem	100 μM
LY294002	PI3K	LC Laboratories	20 μM
MK-2206	AKT	Selleckchem	1.25 μM
U73122	PLCγ	Selleckchem	4 μM

To measure UL138 transcription differences during CD34 long-term culture, CD34^+^ cells were infected with 2 MOI of either WT or ΔEGR1_Site1_ mutant TB40/E_GFP_. At 24 hpi, samples were sorted for CD34^+^ and GFP^+^ cells to get a pure population of infected cells. Cells were seeded into transwells over a feeder layer of irradiated SL/SL and M2-10B4 stromal cells (Stem Cell Technology) in Myelocult (Stem Cell Technology) with pen/strep and 1 μM of hydrocortisone. After 5 days, RNA was isolated, cDNA synthesized, and *UL135* and *UL138* transcripts were quantified with gene specific primers (Table 1), as described above. Because *UL135* transcripts also encode *UL138*, a ratio of UL138 to UL135 transcripts was calculated using Pfaffl to assess changes between WT to mutant virus ($Ratio=\frac{{{Eff}_{UL138}}^{\left({CT}_{\left(WT\;UL138\right)}-{CT}_{\;{(\Delta EGR1}_{Site\;1}UL138)}\right)}}{{{Eff}_{UL135}}^{\left({CT}_{\left(WT\;UL135\right)}-{CT}_{\;({\Delta EGR1}_{Site\;1}UL138)}\right)}}$) [83].

### Immunofluorescence

Samples were processed as previously described and stained with antibodies (Table 2; [84]). All images were obtained using a DeltaVision RT inverted Deconvolution microscope. Representative single plane images were adjusted for brightness and contrast.

### EGF pulse

Uninfected or cells infected with WT or mutant TB40/E_GFP_ virus were washed two time with PBS and serum starved in serum-free media for 24h prior to EGF stimulation. After serum starvation, cells were washed with ice cold PBS and incubated on ice for 30 min. Cells were then incubated on ice with serum free media containing 10 nM EGF (Gold Biotechnology) for 30 min, except for no EGF control. Cells were then washed with ice cold PBS. 37°C serum free media was added and samples were incubated at 37°C for 15min to 24h, depending on experiment. Samples were then collected for immunoblotting or chromatin immunoprecipitation, depending of experiment.

### siRNA knockdown

HEK293T cells, seeded into 12-well plates the day before, were co-transfected with the indicated 400ng pGEMT plasmid and 600ng pSiren plasmid per well using Lipofectamine 2000 (Invitrogen). 24 h later, the cells were serum starved overnight in 0% FBS DMEM and then treated with 50 ng/mL EGF (Cell Guidance Systems) for 1 hour. Cells were harvested in protein lysis buffer (50mM Tris-HCl pH 8.0, 150mM NaCl, 1% NP-40, and protease inhibitors). The experiment was performed in duplicate.

### Measurement of infectious virus

Confluent fibroblasts were infected with either 1 MOI or 0.02 MOI of either WT TB40/E_GFP_ or EGR1 mutant virus (TB40/E-ΔEGR1_Site 1_ and TB40/E-ΔEGR1_Site 2_). For pathway inhibitors, the media was changed 24 hpi with media containing inhibitor and incubated for 8 days. For EGR1 mutant virus studies, media was changed 24 hpi and samples were collected up to 16 dpi. Both cells and media were collected and then total virus was quantified by the TCID_50_ [17]. Infectious centers were quantitated in CD34^+^ HPCs, as described previously [84]. Frequency of infection centers were calculated using extreme limiting dilution analysis [85]. For pathway inhibitors, CD34^+^ HPCs were treated with chemical inhibitors after sorting for CD34^+^ GFP^+^ populations and when stromals were replaced at 6 dpi. Inhibitor concentration, targets, and sources are listed in Table 3. Proliferation of CD34^+^ cells during chemical inhibition was calculated by observing the fold change in the number of cells prior to and after inhibition for each condition.

### Next generation sequencing analysis

Transcript data was acquired from a previous study conducted by Cheng et al. [2]. Briefly, they prepared mRNA libraries were prepared from CD34^+^ HPCs infected with TB40/E_GFP_ at a MOI of 2 at 2 and 6 dpi. Transcripts for EGR1, EGR2, EGR3, and WT1 were normalized to fragments per kilobase per million reads (FPKM) and then normalized to EGR1 levels at 2 dpi. Data from two independent experiments using different donors were combined and graphed together.

### ChIP-qPCR

For EGR1 overexpression chromatin immunoprecipitation coupled with qPCR (ChIP-qPCR), MRC-5 fibroblasts were transduced with 1 MOI of EGR1_3xFLAG_ lentivirus. Transduced cells were then infected with 1 MOI of WT TB40/E_GFP_ for 48h and then processed for ChIP. In EGF pulse ChIP-qPCR, fibroblasts were infected with 1 MOI of either WT TB40/E_GFP_ or EGR1 binding mutant virus,TB40/E-ΔEGR1_Site 1_ or TB40/E-ΔEGR1_Site 2_,and then serum starved at 24 hpi. At 48 hpi, samples were pulsed with 10 nM EGF for 1h. All samples were then processed for ChIP-qPCR using SimpleChIP Enzymatic Chromatin IP Kit (Cell Signaling Technologies) as per manufacturer’s recommended protocol. Fragmentation of DNA was confirmed by the University of Arizona Genetics Core. For fibroblasts, ChIP was carried out using α-EGR1, α-Histone H3 (positive control), and Normal rabbit IgG (negative control) and with 4 x 10^6^ infected cells per reaction (Table 2). For CD34^+^ HPCs, ChIP was carried out using α-EGR1 and Normal rabbit IgG (negative control) with 2 x 10^6^ infected cells per reaction, and samples were run in parallel with a fibroblasts experiment. PCR was performed with primers specific to EGR1 binding site 1 and site 2 in the *UL135* open reading frame and separated on 2% agarose gel with ethidium bromide (Table 1). qPCR was performed with LightCycler SYBR Mix kit (Roche) and Site 1 Forward and Reverse primers. Relative expression was calculated against a 2% input control ($Relative\;expression=0.02\times 2^{\left({CT}_{2\%\;input}-{CT}_{ChIP}\right)}$). Samples were then normalized to the relative expression of either the IgG control or the WT EGR1 ChIP, with the latter being used for mutant virus comparisons.

### Pathscan antibody array

MRC-5 fibroblasts were infected at an MOI of 1 with TB40/E_GFP_ virus and incubated for 48h. After 48h, samples were washed with PBS twice and either lysed to measure steady state phosphorylation or pulsed with 10 nM of EGF for 30 min and then lysed to measure phosphorylation post stimulation. Either uninfected or TB40/E_GFP_ CD34^+^ HPCs were collected from purified populations maintained in long-term culture for 10 days and lysed to measure phosphorylation levels. Phosphorylation levels were measured using the PathScan EGFR Signaling Antibody Array Kit from Cell Signaling as per manufacturer recommended protocol using protein lysates at a concentration of 1 mg/ml. Arrays were analyzed using a LiCOR Odyssey scanner at a resolution of 42μm, high quality setting, and exposure intensity of 1. Phosphorylation levels were normalized to uninfected, no EGF treatment.

### Flow cytometry based viability assay

Fibroblasts were infected with 1 MOI of TB40/E virus. After 24h, infected cells were then treated with MEK/ERK, PI3K/AKT, STAT1/3, or PLCγ inhibitors for 5 days (Table 3). Cell were collected using trypsin, washed with PBS, and stained using Zombie UV Fixable Viability Kit (Biolegend) as per manufacturers recommended protocol. Cells were then washed and resuspended in 2% formaldehyde in PBS. Samples were measured using a BD LSRII equipped with FACSDiva Software (BD Bioscience Immunocytometry Systems) and overlaid using FlowJo software.

### Statistical analysis

All statistics were calculated using GraphPad Prism version 7 software. Statistics for experiments in this study were calculated using either Student T-test or analysis of variance (ANOVA) for statistical comparison, which is indicated in the figure legends with p-values for each experiment.

## Supporting information

S1 Fig Phosphorylation screen of EGFR signaling pathways during CMV infection. Fibroblasts were infected with TB40/E_GFP_ (MOI = 1) for 48 h. Cells were then stimulated with 10 nM EGF for 30 min and lysed for PathScan EGFR Signaling Antibody Array Kit (Cell Signaling) analysis. Parallel unstimulated samples were lysed for comparison. Phosphorylation levels for EGFR (A), MEK/ERK (B), AKT (C), STAT3 (D), and PLCγ (D) markers were normalized to uninfected, no EGF levels and graphed. Data represents two independent screens each containing two internal technical replicates. Error bars represent the range of the means from each experiment. (E-H) The same markers were quantified in CD34^+^ HPCs were infected with WT TB40/E_GFP_ virus (MOI = 2), a pure population of CD34^+^/GFP^+^ cells were sorted at 24h, and seeded into long-term culture. After 10 days in culture, cells were lysates were also analyzed by PathScan EGFR Signaling Antibody Array Kit (Cell Signaling).
(TIF)

S2 Fig Confirmation of chemical inhibition. Fibroblasts were treated with (A) DMSO, (B) MEK/ERK inhibitors (Binimetinib; SCH772984), (C) STAT (Fludarabine; S3I-201), (D) PI3K/AKT (LY294002; MK-2206), (E) PLCγ (U73122) and lysates were isolated from 1–5 days. Samples were separated by SDS-PAGE and blotted for α-pAKT(S472), α-pERK1/2(T202/204), α-pSTAT3(Y705), and α-Tubulin. Inhibitor protein phosphorylation levels were normalized to DMSO controls.
(TIF)

S3 Fig Analysis of cellular survival in fibroblasts and proliferation in CD34. (A) Fibroblasts were infected with 1 MOI of WT TB40/E virus. At 24 h, cells were then treated with MEK/ERK, STAT1/3, PI3K/AKT, and PLCγ inhibitors. After 5 days, cells were collected and cellular survival was determined using Zombie UV fixable viability kit (Biolegend). Data analyzed with FlowJo software (BD Biosciences) and represented as fluorescent signal off-set overlay. MK-2206 is excluded due to excessive auto-fluorescence in unstained control. (B) To assess impact of inhibitor on infected CD34^+^ cells treated with pathway inhibitor in Fig 3B during long-term culture we compared the counts before and after inhibition during long-term culture for all assays used in Fig 3B. Graph represents fold proliferation and was analyzed for statistical significance by One-Way ANOVA and no treatment was statistically significant compared to DMSO.
(TIF)

S4 Fig Diagram of EGR1 binding site mutation. *UL135* nucleotide sequence was altered in both a pGEM-T virus plasmid and TB40/E_GFP_ bacteria artificial chromosome backbone to disrupt EGR1 binding site 1 (A) and EGR1 binding site 2 (B). Mutations were engineered into the wobble codon in order to alter the nucleotide sequence but not the amino acid sequence of UL135. Binding sequence for each site is underlined and nucleotides mutated are indicated in grey boxes and bolded text.
(TIF)
