# Supplementary figures and images for: Host signaling and EGR1 transcriptional control of human cytomegalovirus replication and latency
Source: PLoS Pathog. 2019 Nov 14;15(11):e1008037. doi: 10.1371/journal.ppat.1008037 (PMC6855412; doi:10.1371/journal.ppat.1008037)

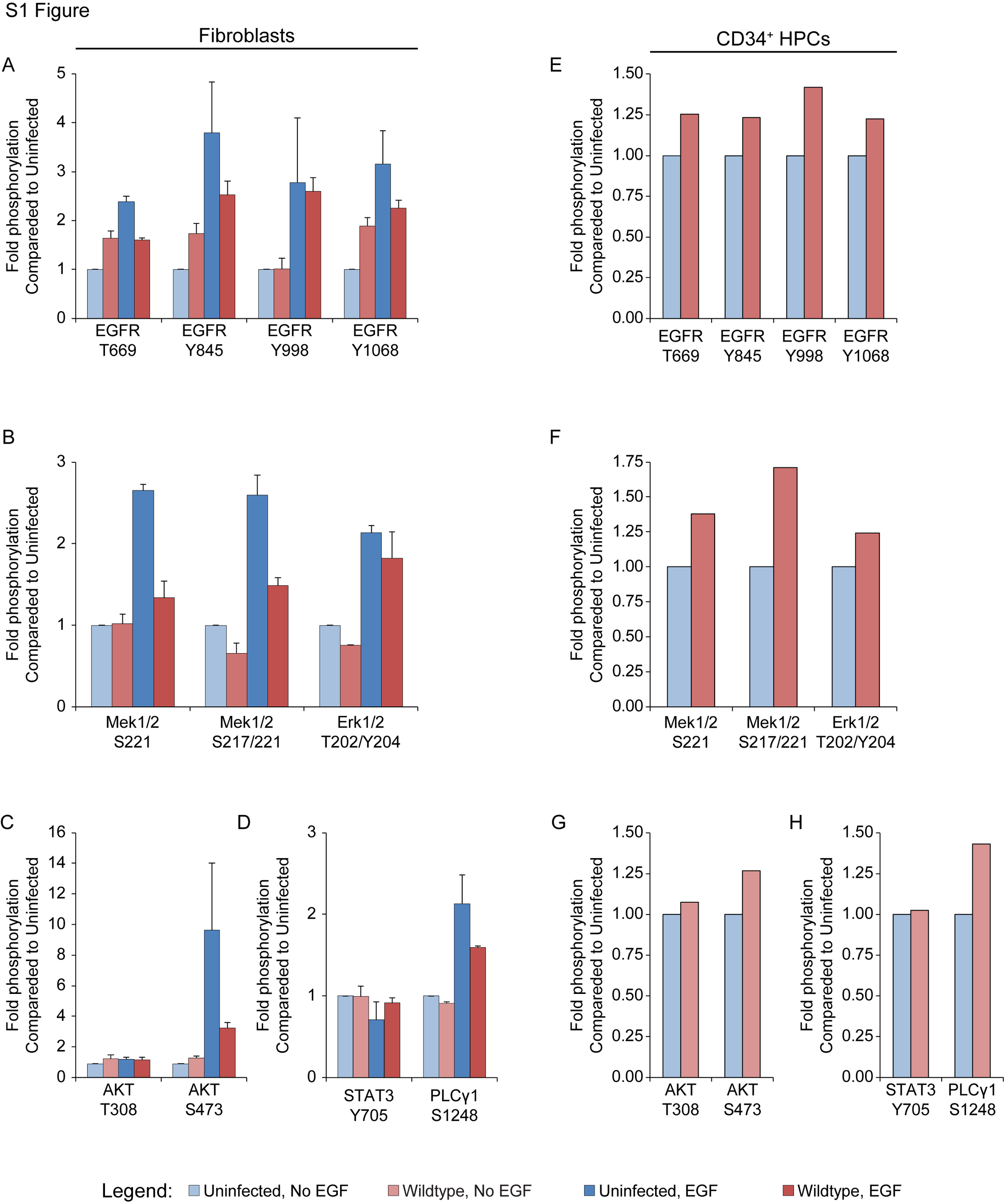

Supplement: S1 Fig — Fibroblasts were infected with TB40/EGFP (MOI = 1) for 48 h. Cells were then stimulated with 10 nM EGF for 30 min and lysed for PathScan EGFR Signaling Antibody Array Kit (Cell Signaling) analysis. Parallel unstimulated samples were lysed for comparison. Phosphorylation levels for EGFR (A), MEK/ERK (B), AKT (C), STAT3 (D), and PLCγ (D) markers were normalized to uninfected, no EGF levels and graphed. Data represents two independent screens each containing two internal technical replicates. Error bars represent the range of the means from each experiment. (E-H) The same markers were quantified in CD34+ HPCs were infected with WT TB40/EGFP virus (MOI = 2), a pure population of CD34+/GFP+ cells were sorted at 24h, and seeded into long-term culture. After 10 days in culture, cells were lysates were also analyzed by PathScan EGFR Signaling Antibody Array Kit (Cell Signaling). (TIF) [file ppat.1008037.s001.tif]

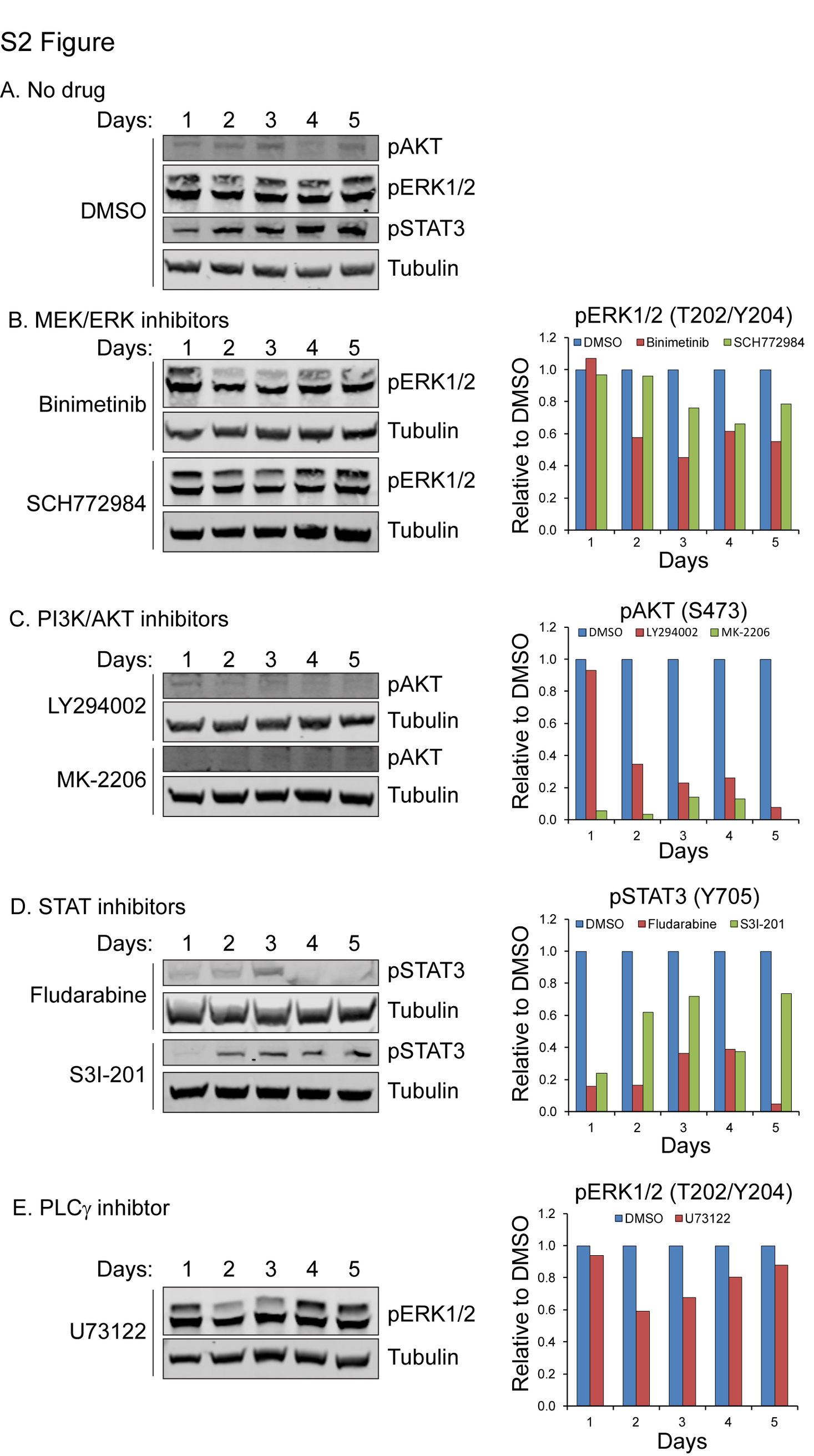

Supplement: S2 Fig — Fibroblasts were treated with (A) DMSO, (B) MEK/ERK inhibitors (Binimetinib; SCH772984), (C) STAT (Fludarabine; S3I-201), (D) PI3K/AKT (LY294002; MK-2206), (E) PLCγ (U73122) and lysates were isolated from 1–5 days. Samples were separated by SDS-PAGE and blotted for α-pAKT(S472), α-pERK1/2(T202/204), α-pSTAT3(Y705), and α-Tubulin. Inhibitor protein phosphorylation levels were normalized to DMSO controls. (TIF) [file ppat.1008037.s002.tif]

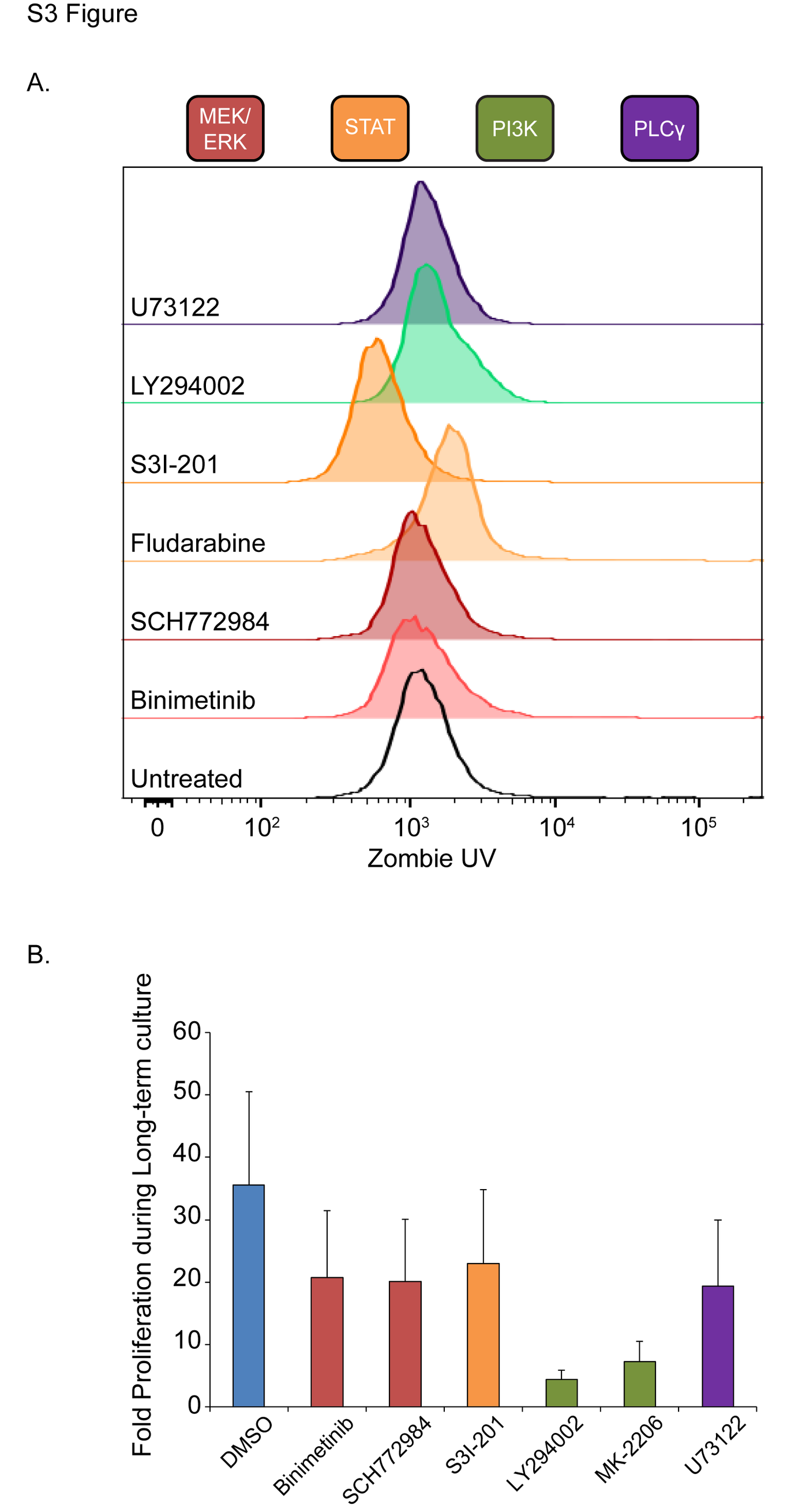

Supplement: S3 Fig — (A) Fibroblasts were infected with 1 MOI of WT TB40/E virus. At 24 h, cells were then treated with MEK/ERK, STAT1/3, PI3K/AKT, and PLCγ inhibitors. After 5 days, cells were collected and cellular survival was determined using Zombie UV fixable viability kit (Biolegend). Data analyzed with FlowJo software (BD Biosciences) and represented as fluorescent signal off-set overlay. MK-2206 is excluded due to excessive auto-fluorescence in unstained control. (B) To assess impact of inhibitor on infected CD34+ cells treated with pathway inhibitor in Fig 3B during long-term culture we compared the counts before and after inhibition during long-term culture for all assays used in Fig 3B. Graph represents fold proliferation and was analyzed for statistical significance by One-Way ANOVA and no treatment was statistically significant compared to DMSO. (TIF) [file ppat.1008037.s003.tif]

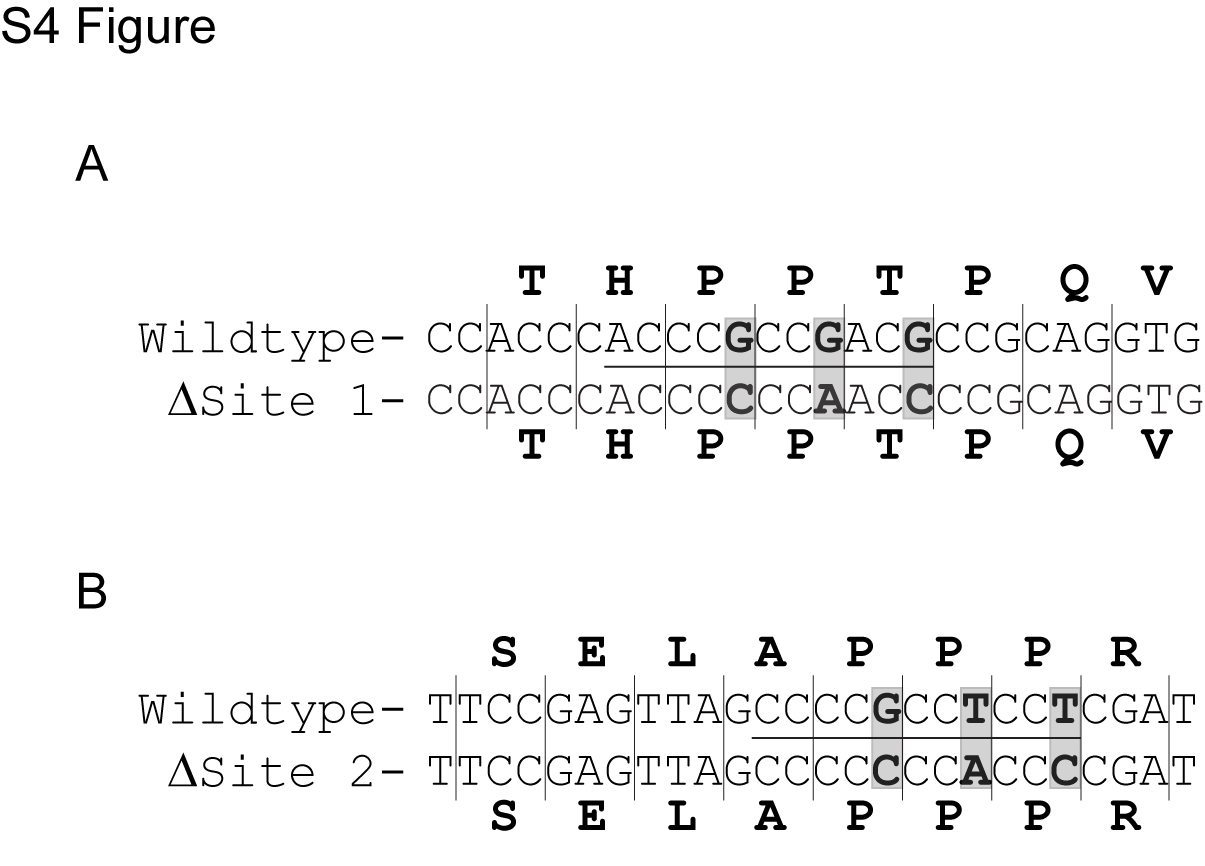

Supplement: S4 Fig — UL135 nucleotide sequence was altered in both a pGEM-T virus plasmid and TB40/EGFP bacteria artificial chromosome backbone to disrupt EGR1 binding site 1 (A) and EGR1 binding site 2 (B). Mutations were engineered into the wobble codon in order to alter the nucleotide sequence but not the amino acid sequence of UL135. Binding sequence for each site is underlined and nucleotides mutated are indicated in grey boxes and bolded text. (TIF) [file ppat.1008037.s004.tif]
